# Assessing biases in phylodynamic inferences in the presence of super-spreaders

**DOI:** 10.1186/s13567-019-0692-5

**Published:** 2019-09-27

**Authors:** Arata Hidano, M. Carolyn Gates

**Affiliations:** EpiCentre, School of Veterinary Science, Massey University, Palmerston North, New Zealand

## Abstract

Phylodynamic analyses using pathogen genetic data have become popular for making epidemiological inferences. However, many methods assume that the underlying host population follows homogenous mixing patterns. Nevertheless, in real disease outbreaks, a small number of individuals infect a disproportionately large number of others (super-spreaders). Our objective was to quantify the degree of bias in estimating the epidemic starting date in the presence of super-spreaders using different sample selection strategies. We simulated 100 epidemics of a hypothetical pathogen (fast evolving foot and mouth disease virus-like) over a real livestock movement network allowing the genetic mutations in pathogen sequence. Genetic sequences were sampled serially over the epidemic, which were then used to estimate the epidemic starting date using Extended Bayesian Coalescent Skyline plot (EBSP) and Birth–death skyline plot (BDSKY) models. Our results showed that the degree of bias varies over different epidemic situations, with substantial overestimations on the epidemic duration occurring in some occasions. While the accuracy and precision of BDSKY were deteriorated when a super-spreader generated a larger proportion of secondary cases, those of EBSP were deteriorated when epidemics were shorter. The accuracies of the inference were similar irrespective of whether the analysis used all sampled sequences or only a subset of them, although the former required substantially longer computational times. When phylodynamic analyses need to be performed under a time constraint to inform policy makers, we suggest multiple phylodynamics models to be used simultaneously for a subset of data to ascertain the robustness of inferences.

## Introduction

During infectious disease outbreaks, policy-makers need accurate epidemiological information such as the time of disease introduction, basic reproduction number, and transmission chains in order to make appropriate disease control decisions. These parameter estimates can be challenging to obtain from traditional epidemiological survey data collected during outbreak investigations, which are often biased by the sampling strategies and poor population coverage. To overcome this problem, researchers are increasingly using phylodynamic methods to make epidemiological inferences. These methods are based on evaluating the genetic diversities among samples that are collected during the outbreak [1] and have proven particularly useful for fast-evolving pathogens such as RNA viruses [2] as well as other pathogens [3].

The term “phylodynamics” was first coined by Grenfell et al. [4] in 2004 to describe research studies that integrate immunodynamics, epidemiology, and evolutionary biology to better understand the link between epidemic processes and pathogen evolution. Within the context of infectious disease epidemiology, phylodynamic studies can be roughly categorised into two groups based on their purpose, the scale of the study, and the availability of data. The first group aims to determine the so-called “who infected whom” describing the transmission chain among infected individuals. When relatively rich epidemiological information is available, this approach has the potential to identify under-detected infections [5], risk factors associated with transmissions [6], and the index case in the outbreak [7]. A recent study examined the validity of different methods available in this group [8]. The other group of phylodynamic studies focuses on making population-level inferences based on phylogeny or genealogy, such as the historical changes in the effective population size [9, 10], the time of the initial disease introduction [11, 12], and geographical spread patterns [13–17].

Many infectious disease studies that make population-level inferences use a Bayesian framework which offers a great advantage of accounting for uncertainties in evolutionary parameters and phylogenetic trees [18]. The development of user-friendly software such as BEAST [19], BEAST2 [20], and MrBayes [21] has also provided researchers with an access to sophisticated methods [22]. With this background, Bayesian evolutionary analysis has been frequently applied to many of recent important human and livestock disease outbreaks. For instance, Scarpino et al. [23] analysed the genetic sequence data from the 2014 Sierra Leone Ebola outbreak using both the Bayesian evolutionary analysis based on BEAST and transmission chain analysis. The authors reported the estimated starting date of the outbreak were similar in both analyses. Using the 2009 H1N1 influenza A pandemic as an example, Hedge et al. [24] investigated how the temporal window of genetic sequence sampling and its sample size affect the accuracy of real-time estimates of the basic reproduction number under different BEAST models. Boskova et al. [25] conducted a comprehensive analysis on genetic sequence data from ZIKA virus outbreak in Brazil and USA using Bayesian and non-Bayesian methods. In particular, the authors performed a parallel analysis using two different tree priors—the coalescent skyline plot [10] and birth–death skyline model [9]—identifying that phylodynamic inferences including the time to the most recent common ancestor (TMRCA) may be unreliable if genetic sequence data contain few variations. Simulating Salmonella outbreaks between animal and human populations, Bloomfield et al. [26] reported two Bayesian ancestral state reconstruction models were unable to accurately recover known population and transmission parameters used in the simulation.

Bayesian evolutionary analysis of pathogen sequences requires choosing priors for the sequence evolution (substitution model and molecular clock rate) as well as for tree topology and branch length distributions. The use of tree generating models as a prior enables researchers to quantify the relationship between the demographics of genetic population and genetic diversities observed in the population. In this context, population refers to pathogen population and not the host population. The original coalescent theory proposed by Kingman [27], population (or deme) refers to a group of genes where any two genetic sequences have the uniform probability to share a common ancestor. The historical fluctuation of genetic population size, so called effective population size (EPS), is of significant epidemiological interest because EPS may indicate how disease prevalence has changed over time in the past [28, 29]. Commonly used tree generating models include coalescent models [10] and birth–death models [9], but these models make an assumption that the population is panmictic, meaning that a disease spreads through a homogeneous mixing pattern. This assumption, however, is almost always violated in reality for various reasons. Humans and animals in general have structured populations, which restrict their contacts and hence disease transmission patterns. It has been shown that phylodynamic analyses ignoring structured populations can produce an erroneous inference such as false bottleneck signals [30], false decline in EPS towards the present [31], and artificial complex EPS dynamics that do not exist in real [32]. Tree generating models that account for population structures have been developed, however, they still assume homogeneous mixing within each structure [14, 15]. Most recently, the PhyDyn package in the BEAST2 platform made it possible to account for the presence of super-spreaders using the structured coalescent framework [33].

Another notable epidemic characteristic that violates the panmictic assumption is the existence of super-spreaders, which are a small number of individuals in the population who generate a disproportionately large number of secondary cases. This phenomenon can happen, for instance, because some individuals shed a substantially larger amount of pathogen organisms or have higher contact frequencies with other individuals [34, 35]. There has been an increasing interest in understanding how individual heterogenous contact patterns influence phylogenetic trees of pathogens that spread through such a complex contact pattern [36–38]. A recent Swiss study estimated the heterogeneous fitness costs of HIV with different drug mutations [39]. Nevertheless, there is little information as to whether common phylodynamic methods provide reliable estimates when a disease spreads through a heterogeneous contact pattern including situations in which a super-spreader exists. This was therefore the first objective of this study.

Another objective of this study was to evaluate how estimates from analysis using only a subset of available genetic samples would perform compared to that using all available sequences when a disease spreads through a heterogeneous contact pattern. There are two reasons to investigate this. First, with a substantial decrease in the cost of obtaining high resolution genetic data, the number of sequences available for phylodynamic analysis will be expected to only grow. However, phylodynamic analysis for a large dataset is still computationally infeasible. Second, many tree generating models in phylodynamic analysis assume that samples were selected randomly from the background population of sequences during a given time period. For infectious disease studies, this assumption is rarely met because of imperfect diagnostic test sensitivity, incomplete epidemiological tracing due to imperfect movement records, and a time-varying surveillance intensity. The last factor in particular results in a phenomenon called preferential sampling [40], where samples may be more frequently collected from epidemiologically closely linked individuals. Studies showed that phylodynamic analyses provide systematically biased EPS estimates under preferential sampling [41]. A recent study suggested that subsampling sequences stratifying based on time and spatial location may able to reduce this [32]. To the best of our knowledge, however, there is no information how subsampling performs in heterogeneous disease transmission systems. We investigated these two questions using a novel framework which simultaneously simulates genetic mutations and disease spreads over a real livestock movement network.

## Materials and methods

### An overview of study

Using a theoretical disease modelling framework, we first explored how epidemic characteristics affect phylodynamic inferences in the presence of super-spreaders, which infect a large number of others. We created a “truth” dataset where all epidemiological and evolutionary parameters were known using a novel spatially explicit disease simulation model in an individual-based model framework. The model had three components: (1) a demographic component describing various demographic events including between-herd livestock movement, births, deaths, and culling of animals, (2) a disease transmission component describing pathogen transmission between individuals within a herd and between herds, and (3) a genetic mutation component describing nucleotide substitutions over time. Obtained genetic sequences were then used to estimate epidemic starting dates using BEAST analysis. We quantified the degree of bias in estimates and investigated which epidemic characteristics influenced the estimates. Finally, we repeated these analyses under different subsampling strategies and evaluated how these strategies performed compared to when all available sequences were used.

### Disease simulation

#### Demographic component

We used the New Zealand dairy farm network as a sample population for the simulation framework since this represents a large population of approximately 12 000 farms and the contact structure is known to be highly heterogeneous [42]. A data-driven modelling approach was used where demographic events were modelled as a deterministic non-Markov process using data on herd demographics and individual animal events including births, deaths, culls, and movements between farms. Data were extracted from the New Zealand Dairy Industry Good Database (DIGAD), which contains demographic records for approximately 70% of commercial dairy herds in New Zealand over the past 30 years [43]. For the purpose of this study, we used records from 1^st^ July 2000 to 31^st^ June 2010. Herds in a given year were defined as a group of animals under the same ownership that were present between 1^st^ July and 31^st^ June reflecting the typical production season in New Zealand [44]. Birth events represented new calves join the calf group of a given herd, while death or culling events resulted in the animal being removed from a given herd. Details of livestock movement data used can be found in Additional files 1, 2 and 3. When movements data were aggregated over a year separately for each age category, many of these distributions followed power law with exponent 3 to 4 (Additional file 4). Whereas more than 90% of farms had equal to or less than 5 in- and outdegree (number of farms connected), less than 1% of farms had equal to or greater than 30 in- and outdegree (Additional file 5).

We then developed an individual-based metapopulation model with two layers: within-herd and between-herd dynamics. The within-herd dynamics concern the transition of individual animals between management groups in a single herd. For simplicity, we grouped individuals into three age groups within a herd; calves that are ≤ 12 months of age, heifers > 12 and ≤ 24 months of age, and adults > 24 months. We assumed that each age group did not mix with other groups and hence there is no direct disease transmission between different age groups except when animals move between groups due to aging. Calves and heifers moved to the heifer and adult age groups, respectively, on 10^th^ July every year. When animals moved between herds via a livestock movement, they joined the corresponding age group in the destination herd; for instance, adult animals from one farm only move to the adult age group of the destination herd. The geographic locations of herds were divided into 16 geographical regions as previously described for the purpose of informing the detection and sampling strategies described below [42].

#### Disease transmission component

Given that the objective was to create an epidemic scenario in which a super-spreader exists and a disease only transmits through a livestock movement, we used a hypothetical disease which has following characteristics: (1) it is a rapidly mutating pathogen (FMDV-like), providing a relatively large number of genetic mutations in a limited time duration, (2) it spreads between individuals within an age group via a direct contact and between herds only via a movement of infected individuals, (3) it only infects cattle and no other ungulate species, and (4) it does not spread through a local spread (e.g. airborne transmission).

Individuals were classified into one of the following four mutually-exclusive disease statuses: susceptible (S), exposed (E), infectious (I), and immune (R). Where there is at least one infectious animal, each susceptible individual in the same age group is assumed to receive the force of infection λ, which is described as follows:

$$\lambda_{ut} = C_{e} \times I_{ut } \div N_{ut}$$

where $C_{e}$, *I*_*ut*_, and *N*_*ut*_ represent the number of individuals effectively contacted by a given individual per unit time, the number of infected individuals, and the number of total individuals at a given time *t* in a given animal group *u* (i.e. a given age group in a given herd). Note that other studies may use a different expression for the force of infection and we used a formula described in [45]. Individuals in E status subsequently move to I status after a latent period and move from I status to R status after a set infectious period. Each newly infected animal acquires the same genetic sequence as the one that is randomly selected from all genetic sequences carried by infected animals in the same age group in a given herd at the time when the new infection happened.

In the disease transmission component, disease transmission within a herd was modelled as a continuous-time Markov process. However, transitions of individual animal disease status (i.e. transition from E to I and transition from I to R) were modelled as a non-Markov event. This was to avoid the assumption that the probability of an individual animal transitioning to the next disease status is independent of the time the animal had spent in the current disease status, which is unlikely to hold true for many diseases in reality. Values for latent and infectious period were randomly sampled from distributions described in Additional file 6. We arbitrarily chose to use these parameter distributions because they were largely consistent with the values reported for many real diseases and were sufficient to ensure that epidemics were able to take off without becoming extinct too often.

#### Genetic mutation component

As previously explained, each infected animal carried one genetic sequence, but multiple genetic sequences could be present within a single herd. For the sequence, we arbitrary chose a length of 633 nucleotides because similar length was used in previous phylodynamic studies [32, 46]. Once a susceptible animal acquired a pathogen with a given sequence, a nucleotide substitution process started occurring over time. This mutation was assumed to occur following Jukes–Cantor model with a substitution rate of 0.012 per site per year [47] in individuals that have either status E or I. The mutation was modelled as a continuous-time Markov process. We assumed there was no recombination between sequences and individuals can be infected by only one virus. As a genetic sequence for the initial case, we arbitrary chose the sequence of Genbank Accession number FJ785304.1 [48].

#### Disease detection and control component

Disease detection was assumed to occur only via clinical detection when the animal entered the infectious (I) period and not by other means such as slaughterhouse inspection. Each herd had a probability to be detected positive as soon as there was at least one animal in infectious (I) status present in either of three age groups. When there are no farms that are detected positive, we assumed that all infected herds have the same baseline probability to be detected (P_base_, see Additional file 6). To mimic the real situation of disease outbreaks in which resources are concentrated into the areas where detected herds exist, we assumed that infected herds in the geographical regions which have detected herds have an elevated probability of being detected (P_inc_). Once a herd was detected positive, several events occurred. First is the genetic sampling. One animal (hence one genetic sequence) was randomly sampled from all animals that were in infectious (I) status on the herd, only if this herd was never detected positive before. The identification number of a sampled animal, the herd identification number of the herd this animal existed, and the time of detection were recorded. Second was a placement of a movement restriction; the detected herd is banned for moving off any animals for 180 days. For the sake of simplicity, we assumed that farms which purchased animals from now-banned farms in the explicit movement data set would not buy animals from alternative farms. We did not consider any additional epidemiological investigations triggered by a detection such as a backward and forward contact tracing. After 180 days of a movement restriction, all animals in either E or I status were assumed to move into R status.

#### Simulation algorithm and conditions

We used a simulation algorithm that has previously been implemented by other studies [49, 50]; whereas non-Markov events occurred at a given scheduled day, Markov events could occur at any given continuous time. As already explained, there are three Markov events in this simulation; disease transmission, disease detection, and genetic mutation. For Markov events, the cumulative distribution function of inter-event time $\tau$ (i.e. time to a next Markov event: t_MARKOV_) can be described as $q\left( \tau \right) = 1 - e^{{ - {\text{B}}t}}$, where *B*_*t*_ represents the sum of the rate of all Markov events at a given time *t*. By randomly sampling a value from this distribution, we can calculate the t_MARKOV_ at the time *t*. On the other hand, for non-Markov events, the time at which these events occur are pre-defined, therefore, we also know the time to a next non-Markov event, t_NON-MARKOV_. We calculated t_MARKOV_ every time when any Markov and non-Markov events occurred. Calculated t_MARKOV_ was then compared to t_NON-MARKOV_, and if t_NON-MARKOV_ < t_MARKOV_ the non-Markov event was carried out and we again calculated the time for the next Markov event: otherwise, the Markov event was implemented and the simulation day was updated.

To ensure that disease spread to a large enough number of farms and hence a sufficient number of genetic sequences could be collected, we chose one herd randomly from farms that moved at least one adult animal off to at least seven other farms within the 1^st^ year as the infection seed on Day 0 (which is 1^st^ July 2000). There were 109 farms with 12 213 adult animals that met this condition. At the chosen herd, one animal from each of three age groups was randomly chosen to be infected. Their disease statuses were set to I and allocated the genetic sequence that was described in “Genetic mutation component” section. Furthermore, we assumed that the first disease detection would not occur by Day 365 to prevent an epidemic from going into extinct too quickly.

At each iteration, we recorded following information for all infected animals; animal identification number, genetic sequence identification number, identification number of sequence that infected this animal, infection date, identification number of farm at which this animal was infected, whether or not this sequence was isolated, and nucleotide sequence if isolated. Isolated sequences were then subsampled according to sampling strategies described below and exported as a NEXUS file, which is the required format for BEAST analysis. The simulation model was coded in the C language and the simulation codes were rigorously checked for its validity. The model validation process and reproducible codes can be found at [51]. The simulation code can be found at the first author’s repository [52]. In each iteration, a simulation was run until one of the following three conditions was met: (1) the total simulation duration reached 10 years, (2) until the disease died out, or (3) a total of 150 genetic sequences were obtained. Results of iterations were only analysed when at least 50 genetic sequences were obtained—this is to avoid a sample size being too small after the subsampling process for the Phylodynamic analysis. This process was repeated until a total of 100 eligible epidemic iterations were obtained.

#### Definition of super-spreaders

A definition of super-spreaders is relative and can vary in each epidemic and for different diseases. Given our simulation framework uses a stochastic, data-driven model we did not explicitly model super-spreader; rather, we defined them post hoc based on the farm’s effective production number (R), which was calculated in each simulation. We initially defined a farm as super-spreader when the farm had R ≥ 20, and varied this cut-off value (10, 15, 30, and 40) to investigate the impact of the cut-off value.

### Descriptive analysis of epidemics

A farm-level transmission network was created for each of 100 simulated disease spread to calculate various summary statistics of their epidemic characteristics. Although we knew exactly who infected whom on an individual animal level, we chose to describe the disease transmission on a farm level because the latter is of interest in many epidemic situations. Two types of variables were computed; variables related to super-spreader and those related to other epidemic characteristics. Variables related to super-spreader included (1) R calculated as the average number of secondary cases generated by each infected farm, (2) their standard deviations, (3) the maximum R divided by the total number of infected farms (hereafter, max R proportion), which indicates how dominant a super-spreader was in the epidemic, (4) max R proportion calculated after excluding index farm, (5) whether or not a super-spreader with different R cut-offs (10, 15, 20, 30, and 40) existed, and (6) same measure as (5) but after excluding index farm. Variables related to other epidemic characteristics included (7) whether the index farm was sampled (8) the average shortest path lengths (defined as the number of farm-level transmission events separating any two given infected farms) between all infected farms or (9) between all sampled farms, (10) the average shortest path lengths from the index farm to all infected farms or (11) to sampled farms, (12) epidemic duration in days, (13) number of infected farms, (14) the proportion of infected farms sampled, and (15) normalised Sackin index. The normalised Sackin index represents an imbalance of tree such as phylogenetic tree [53] and transmission tree [54]. We computed the normalised Sackin index $I_{s}$ using the following formula [53]:

$$I_{s} = \frac{{I_{s}^{n} - E\left[ {I_{s}^{n} } \right]}}{n}$$

where, n is the number of tree leaf (a farm which did not infected any farms) and $E\left[ {I_{s}^{n} } \right]$ is the expected Sackin index under the Yule model. The larger a deviation of the normalised Sackin index from 0, the more a transmission tree is imbalanced. The tree imbalance measures a global clustering in the epidemic [54]. Two exemplar transmission trees are shown in Additional file 7.

### Phylodynamic analysis

The obtained genetic sequences were first analysed for the temporal signal using TempEst version 1.5.1 [55] based on maximum likelihood (ML) trees constructed by the R package phangorn version 2.5.5 [56]. The R squared value of a linear regression model between the divergence from the tree root and sampling times was obtained by minimising the mean of the squares of the residuals and used as a measure of the temporal signal strength. As benchmark, we estimated the most recent common ancestor (TMRCA) using least-squares dating methods implemented by the LSD software version 0.3 beta with options -c, -r [57], based on the ML trees constructed as above. Subsequently, TMRCA was estimated using two tree generating models: an Extended Bayesian Coalescent Skyline plot (EBSP) model [58] using BEAST version 1.8.4 [19] and Birth–death skyline plot (BDSKY) model [9] using BEAST version 2.5.2 [20]. In our study, TMRCA approximates to the epidemic duration (T) because we used a single sequence as a seed; at the start (Day 0) there were not multiple sequences that had a common ancestor before Day 0. The date that each sequence was obtained was used as a tip date. The Jukes–Cantor model was used as substitution model assuming a strict molecular clock, and a prior for the substitution rate was set to have a uniform distribution of 0.00002 and 0.00005 per site per day as a lower and upper limit, respectively. We used this narrow prior to identify the mere influence of epidemic characteristics on phylodynamic inferences rather than accounting for an uncertainty around the substitution rate. In BDSKY model, we set the sampling proportion to be 0 before the oldest sampling was collected to avoid bias in the estimates. The detailed settings for BDSKY model can be found in Additional file 8. Other parameters were set to the BEAST default. Markov chain Monte Carlo (MCMC) chain was run over 50 000 000 iterations, sampling at every 1000 iterations, and the first 10% was discarded as a burn-in period. Results obtained by BEAST were summarised using Tracer version 1.7 [59]. The effective sample size (ESS) for each parameter was ensured to be larger than 200. When the ESS was lower than 200, we ran an additional 50 000 000 iterations and examined the ESS again. We repeated this process until the ESS of 200 was achieved.

### Evaluation of phylodynamic inferences

The point estimate from the LSD or the median estimate from Bayesian analysis for the tree height (i.e. TMRCA) was compared to the corresponding known epidemic duration (T) in each epidemic. Following the previous study [32], we calculated two metrics (percent bias and percent error) for accuracy and one for precision (highest posterior density size, only for Bayesian analysis):

$${\text{Percent bias}} = 100 \times \frac{T - estimated\;TMRCA}{T}$$

which represents in which direction and how much the estimate is biased. The negative of this statistic indicates that the TMRCA is overestimated (i.e. the epidemic starting date is estimated too early in the past) and vice versa.

$${\text{Percent error }} = 100 \times \frac{{\left| {T - estimated\;TMRCA} \right|}}{T}$$

which represents the divergence of the estimated median value from the true value.

We also obtained the size of highest posterior density (HPD) as an indicator of the precision of the estimate,

$${\text{HPD size }} = 100 \times \frac{{TMRCA_{97.5} - TMRCA_{2.5} }}{T}$$

where TMRCA_97.5_ and TMRCA_2.5_ represent the upper and lower limit of its 95% HPD. Above three statistics were obtained for each of 100 eligible epidemics. In addition, we calculated two additional statistics: coverage and convergence rate. The former represents the proportion of epidemics (out of 100) in which the HPD contained the true T. The latter represents the proportion of epidemics in which 200 ESS was obtained for parameters of interest within 50 000 000 MCMC iterations as a crude measure of the efficiency of mixing. Note that we run a longer chain for epidemics which did not converge within 50 000 000 iterations as described above.

### Associations between phylodynamic inferences and epidemic characteristics

Associations between the performance of the BEAST inferences and epidemic characteristics were investigated using a linear regression model. Separate models for each of the two outcomes—the percent error and the HPD size—from EBSP and BDSKY model were built using the 15 variables described in “Descriptive analysis of epidemics” section. The percent bias was not investigated because there was no reason to believe the direction of bias (negative or positive) is meaningful in this analysis. Only variables that were significant at an alpha level 0.05 in the univariable analysis proceeded to the multivariable analysis. A multivariable model was constructed by adding one variable at a time in the order of the strength of the association in the univariable analysis using a forward manual approach. Variables significant at an alpha level 0.05 were kept in the final model. We did not assess interactions between variables as the sample size was limited. All the statistical analyses were performed using R version 3.4.2.

### Subsampling strategy

**Figure 1:**
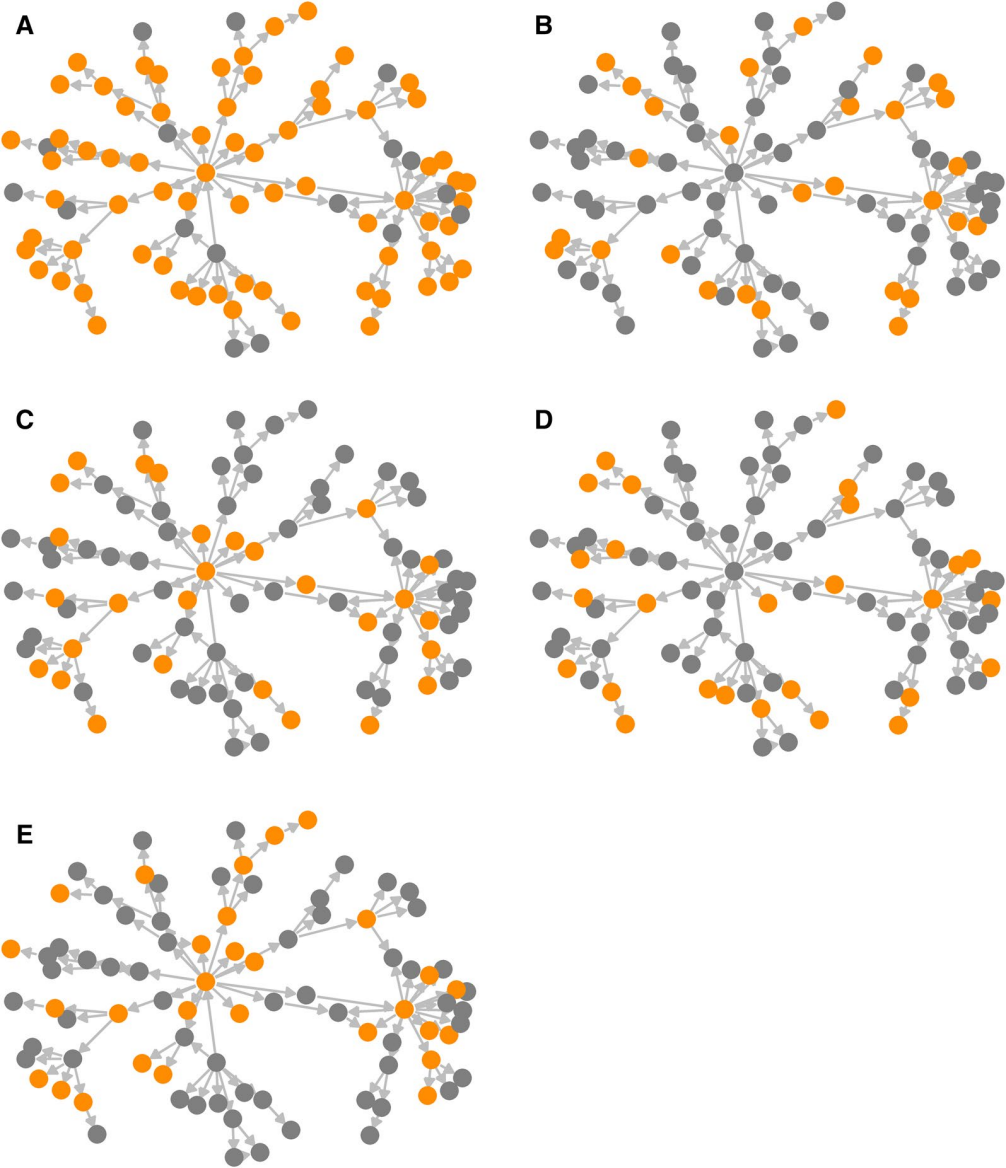
**An exemplar of subsampling strategies evaluated in this study. A** All, **B** Equal, **C** Herd infected, **D** Random, and **E** Herd strategy. Equal number of genetic samples were collected for each subsampling strategy.

To evaluate whether using a subset of genetic samples influences phylodynamic inferences, sampled sequences were further subsampled based on different sampling strategies, mimicking subsampling protocols that are feasible in reality. These strategies include: (1) uniform probability sampling with respect to both time and geographical areas (hereafter referred to as “Equal” strategy), (2) random sampling (“Random” strategy) that does not account for time and geographical areas of samples, (3) random sampling proportional to the number of infected herds in each geographical area (“Herd infected” strategy), and (4) random sampling proportional to the number of herds in each geographical area (“Herd” strategy) so that consistent proportion of herds are collected across regions. In the “Equal” strategy, samples were stratified based on the time and locations and one sample was included from each stratum as follows. First, it was necessary to divide the sampling period into intervals. This sampling period (*T*_*samp*_) corresponds to the period between the earliest and last sampling time. To determine how many sampling intervals (*n*_*i*_) we needed to create, the number of geographical regions (*n*_*r*_) that had at least one sequence-isolated herd was obtained. Based on the total number of genetic sequences collected (*n*_*t*_), *n*_*i*_ was determined as *n*_*i*_ = *n*_*t*_/*n*_*r*_ + 1. This was to maximise the number of samples included in each geographical area while ensuring the probability of selecting a sample in each region was the same. Then, the width of the sampling interval can be described as *T*_*samp*_/*n*_*i*_. We selected one sample per interval per geographical area, which resulted in the sample size of k. For the strategy “Random”, k samples were subsampled without replacement from all collected sequences without any regards to time or geographical regions. For the strategy “Herd”, k samples were selected from each geographical region proportional to the number of herds in each region. As the number of herds, we excluded herds that were not involved in any livestock movements (inactive herds) because they could not be infected in this simulation framework. Given the majority of eligible iterations stopped before Day 1095 (i.e. 3 years from the start), we defined inactive herds as those had no animals that moved in or moved off from the herd between Day 0 and 1095. For the “Herd infected” strategy, k samples were selected from each geographical region proportional to the number of farms that were detected positive in each region. Note that the number of farms detected positive is not necessarily the number of farms infected in each region, but it is often only the information available in the real disease outbreak situation. An exemplar of subsampling strategies was shown in Figure 1.

For each of subsampled data, we repeated the phylodynamic analysis using EBSP model described in “Phylodynamic analysis” section to estimate the TMRCA. The distributions of three statistics (percent bias, percent error, and HPD size) were obtained and compared with those of the scenario in which all available genetic samples were used (hereafter referred to as “All” strategy) in the phylodynamic analysis. The Kolmogorov–Smirnov test was carried out to investigate whether two given distributions were drawn from the same distribution. We also tested whether one of subsampling strategies constantly provide a smaller percent error than the other using Wilcoxon signed-rank test. We did not adjust *p*-value for multiple comparisons because it was deemed less meaningful to carry out a formal statistical evaluation to determine which subsampling strategies perform better when the number of simulated outbreaks was limited to 100.

## Results

### Epidemic characteristics

**Figure 2:**
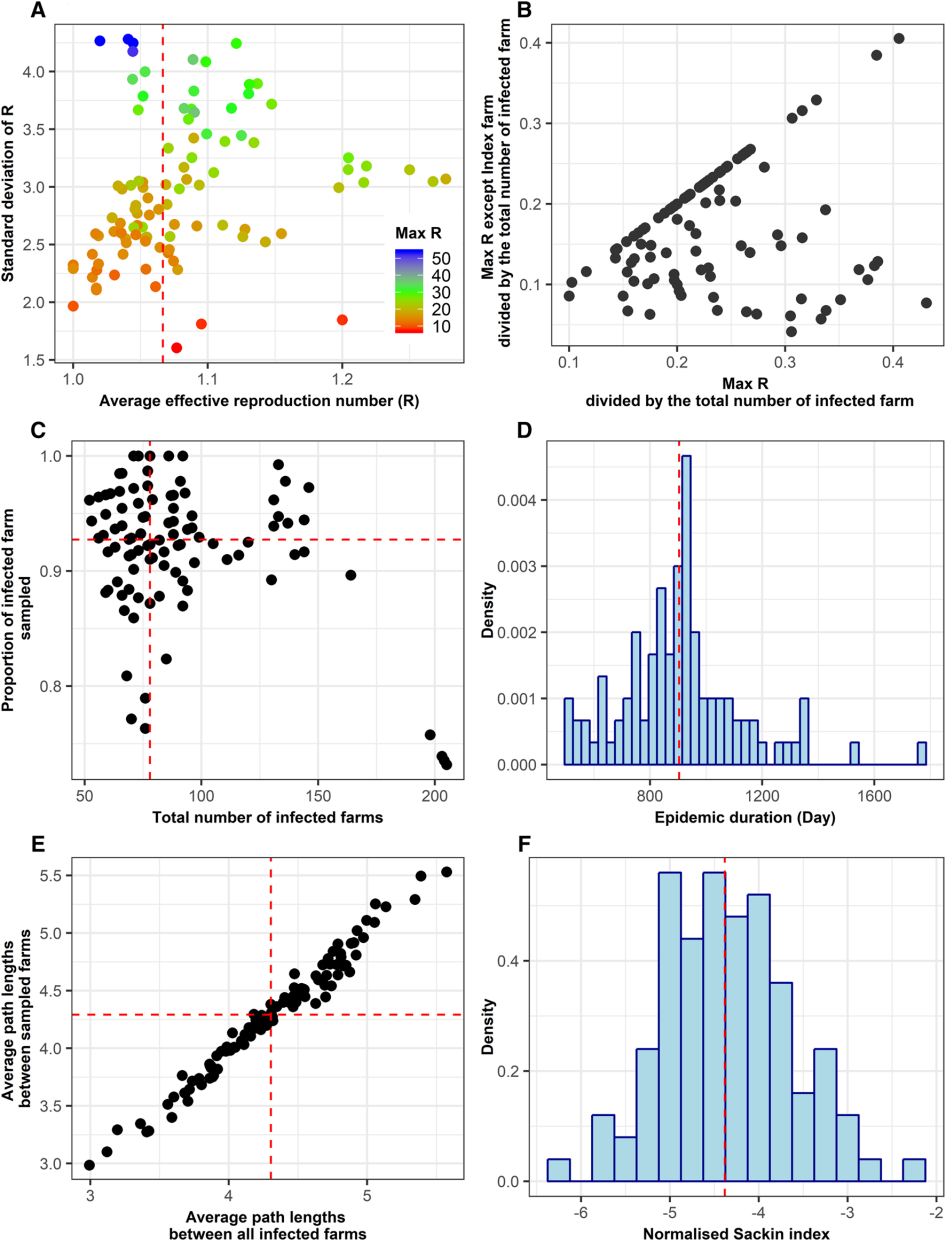
**Descriptive statistics of epidemic characteristics over 100 simulations**. The average number of secondary cases per one infected farm (effective reproduction number; R) and its standard deviation in each of 100 simulated epidemics are shown in (**A**). A high standard deviation indicates a presence of super-spreaders. The colour represents the maximum R in each epidemic.

Figure 2 shows descriptive statistics of 100 simulated epidemics. The median farm-level average effective production number (R) was 1.067. Even though the average R was close to 1, one or more farms generated a substantially large number of secondary cases (maximum 54, Additional files 9 and 10), and these farms may or may not be an index farm as shown in Figure 2B. When super-spreader is defined to be a farm with R ≥ 20, all super-spreaders were found to have exported animals to ≥ 19 farms annually at least one in the first 2 year of the epidemic. The detailed characteristics of super-spreaders identified can be found in Additional files 11, 12 and 13.

The median number of infected farms was 78 (minimum 52 and maximum 205). The median number of infected farms sampled (hence the number of genetic sequences) was 72.5. The proportion of farms sampled out of the total infected farms ranged from 73.2 to 100% and this proportion was larger than 90% in 50 out of 100 simulations. The median value of epidemic duration (which was equivalent to the time to the most recent common ancestor (TMRCA) in this study) was 904 days (minimum 500 days and maximum 1758 days). Detailed descriptive statistics of disease simulation results can be found in Additional files 1 and 14.

### Accuracy and precision of TMRCA estimates using all available samples

**Figure 3:**
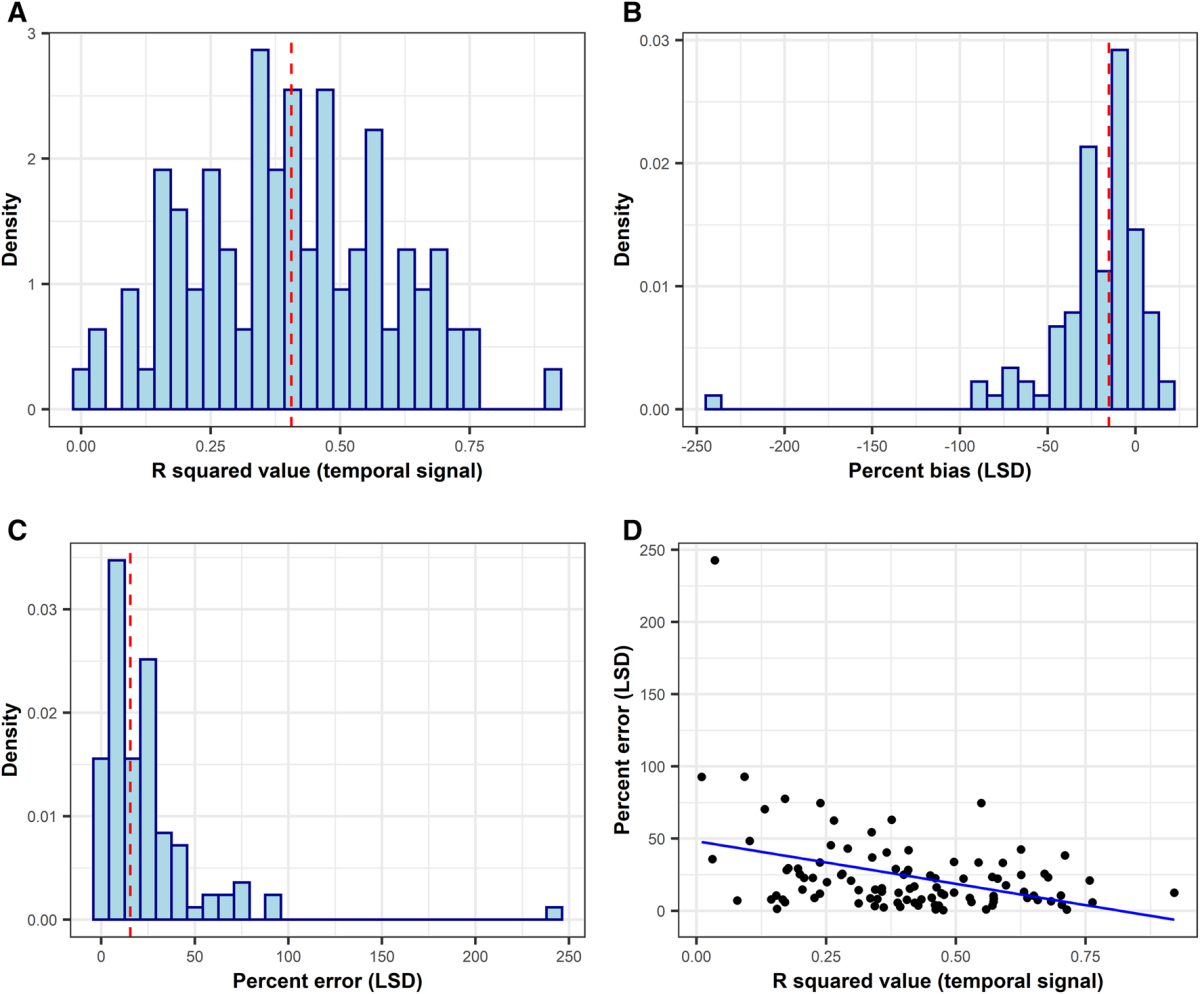
**Results of the least-squares dating (LDS) methods and distribution of the degree of the temporal signal contained in the simulated genetic data.**

**Figure 4:**
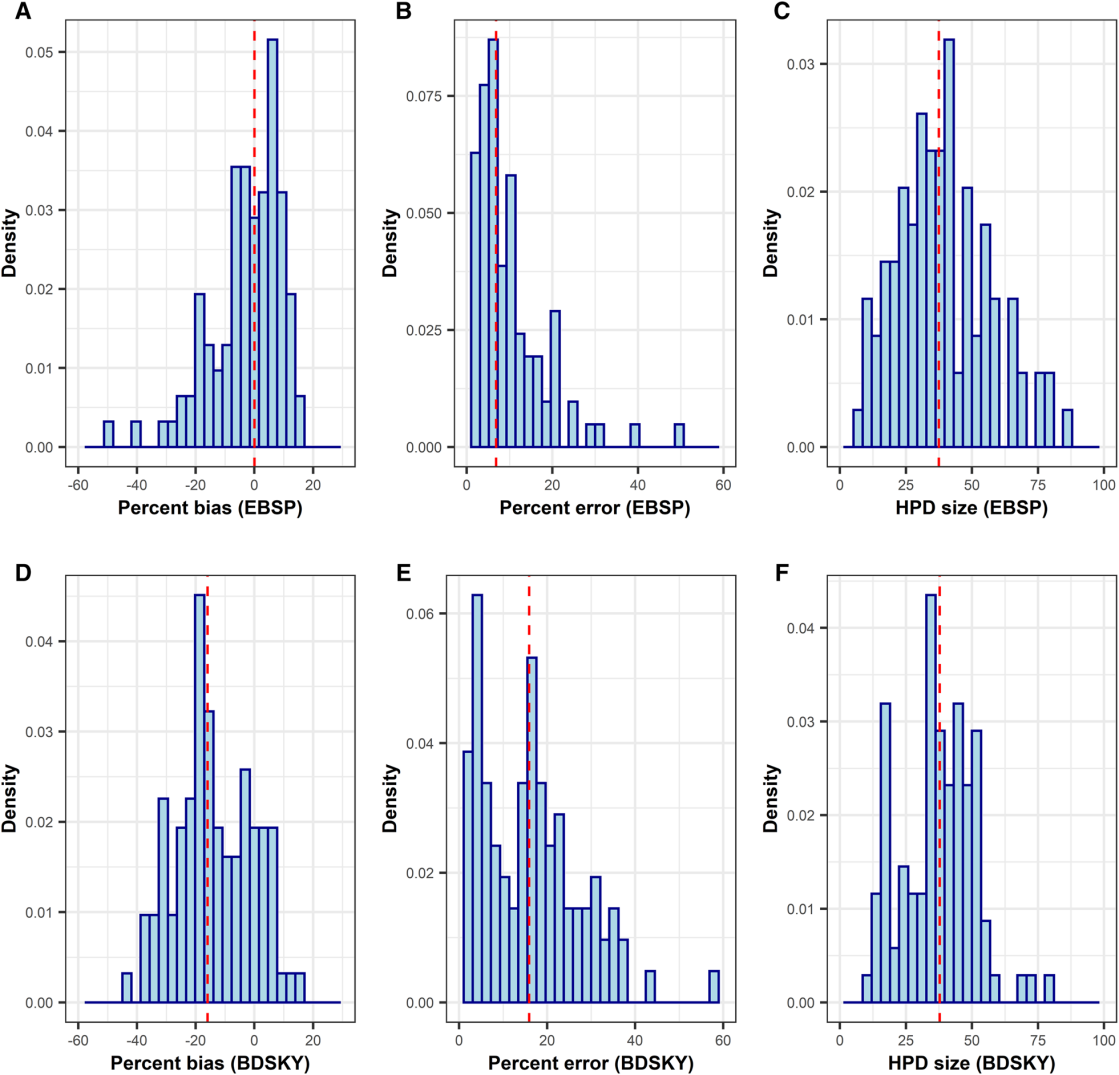
**Accuracies and precision of phylodynamic inferences from EBSP and BDSKY model using all available genetic sequences.**

Figure 3A shows the distribution of the temporal signal contained in the sequence collected, suggesting a large variation between outbreaks. The median percent error in the TMRCA estimate using the LSD method was 15.5, with this value being ≤ 10 and ≤ 50 in 34 and 90 outbreaks, respectively (Figures 3B and C). There was a negative correlation between the R squared value and the magnitude of the error (Figure 3D, correlation coefficient = −0.38, *p* = 0.0001). Figure 4 shows the accuracy and precision of TMRCA estimates by EBSP and BDSKY model when all available genetic sequences were used for the analysis. The minimum and maximum values for percent bias were −49.8 and 15.6 for EBSP and −58.6 and 14.4 for BDSKY, suggesting that an extreme overestimation of TMRCA can occur. The median percent error was 6.87 for EBSP and 15.9 for BDSKY. There was no statistically significant correlation between the degree of the temporal signal and the percent error of both EBSP and BDSKY models (Additional file 15). The median HPD size was 37.5 for EBSP and 37.8 for BDSKY. The accuracy and precision between EBSP and BDSKY strongly correlated. By contrast, the accuracy of the LSD did not show any statistical correlations with either of EBSP or BDSKY (Additional file 16). Estimates on other BDSKY parameters can be found in Additional file 17.

### Association between phylodynamic inferences and the presence of the super-spreader

**Figure 5:**
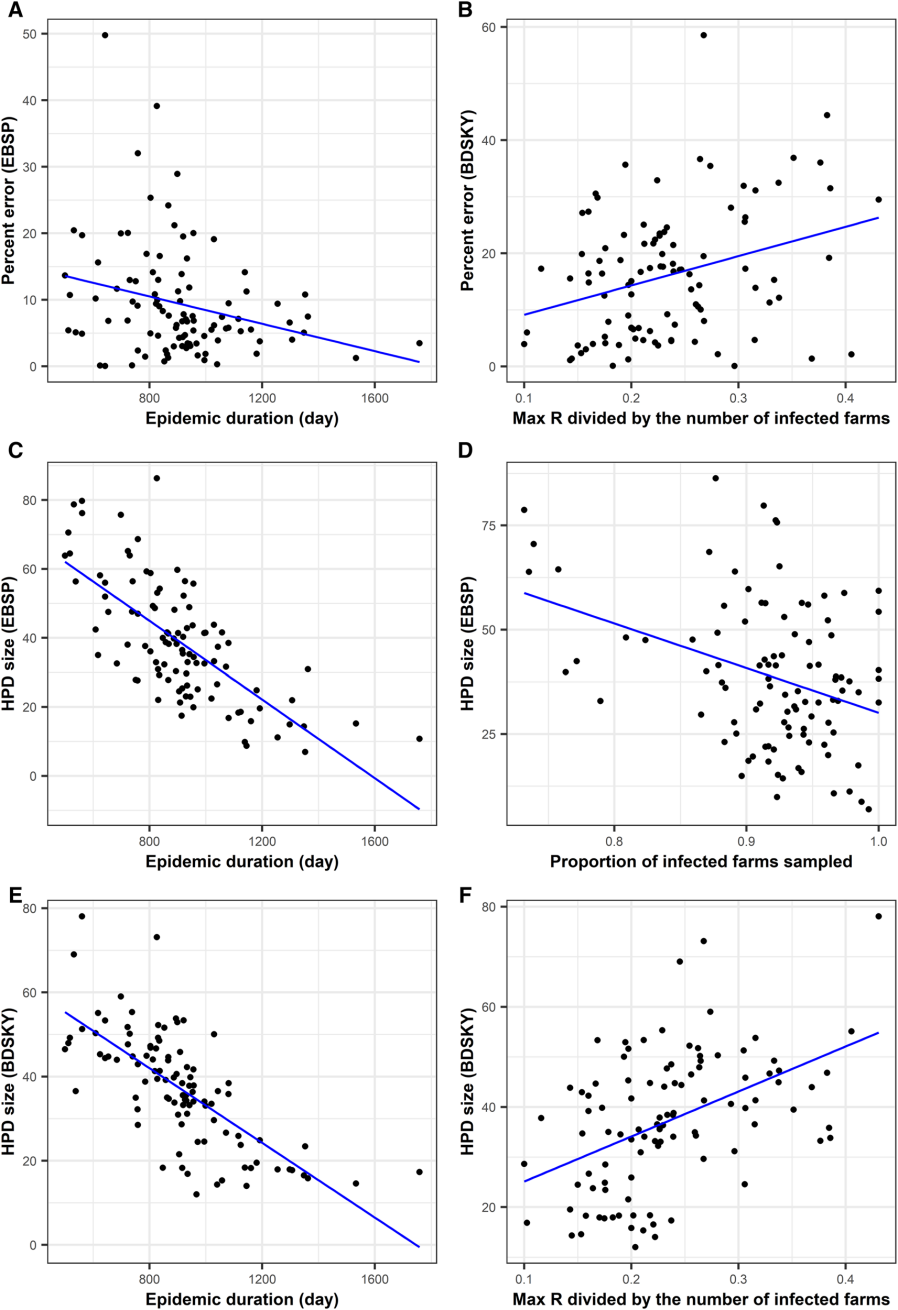
**Associations between super-spreader and epidemic characteristics, and phylodynamic inferences from EBSP and BDSKY model.** The panel (**A**) and (**B**) show the accuracy of phylodynamic inferences (percent error) over 100 simulated outbreaks, whereas (**C**) to (**F**) show the precision of inferences (95% highest posterior density (HPD)).

Among six variables that related to the presence of the super-spreader, none of them were associated with the percent error from EBSP model (Additional file 18). By contrast, the standard deviation of effective production number (R), max R divided by the total number of infected farms (max R proportion), and the presence of farm with R ≥ 20 were univariably associated with the percent error from BDSKY model (Additional file 19). Among these, only max R proportion remained significant in the multivariable linear model after controlling other epidemic characteristics described later (Figure 5).

The HPD size was univariably associated with standard deviation of R, max R proportion, and the presence of farm with R ≥ 40 in both EBSP and BDSKY model (Additional files 20 and 21). Whereas max R proportion remained significant in the multivariable model for the HPD size of BDSKY (Figure 5), none of variables related to super-spreader remained significant in the multivariable model for the HPD size of EBSP (see multivariable model results for Additional file 22).

### Association between phylodynamic inferences and other epidemic characteristics

Among variables related to other epidemic characteristic, “average path lengths between all infected farms”, “average path lengths between all sampled farms” and “epidemic duration in days” showed statistically significant univariable associations with the percent error of EBSP model (Additional file 18). The first two variables were strongly correlated in this study (Figure 2) and both effects disappeared after controlling for epidemic duration (Figure 5, Additional file 22). For BDSKY model, while several variables showed a univariable association with the percent error (Additional file 19), none of them remained significant after controlling for max R proportion as described earlier (Figure 5).

For the HPD size of EBSP, although several variables showed univariable associations (Additional file 20), these effects disappeared after controlling for either epidemic duration (Figure 5C) or proportion of infected farms sampled (Figure 5D). The proportion of infected farms sampled showed a marginal negative association with the HPD size after adjusting by epidemic duration (Additional file 22). Similarly, for the HPD size of BDSKY, only max R proportion (Figure 5F) and epidemic duration (Figure 5E) remained significant in the multivariable model (Additional file 21). The longer an epidemic was, the smaller the HPD size was both for EBSP and BDSKY models.

### Performance of subsampling strategies compared to using all available samples

**Figure 6:**
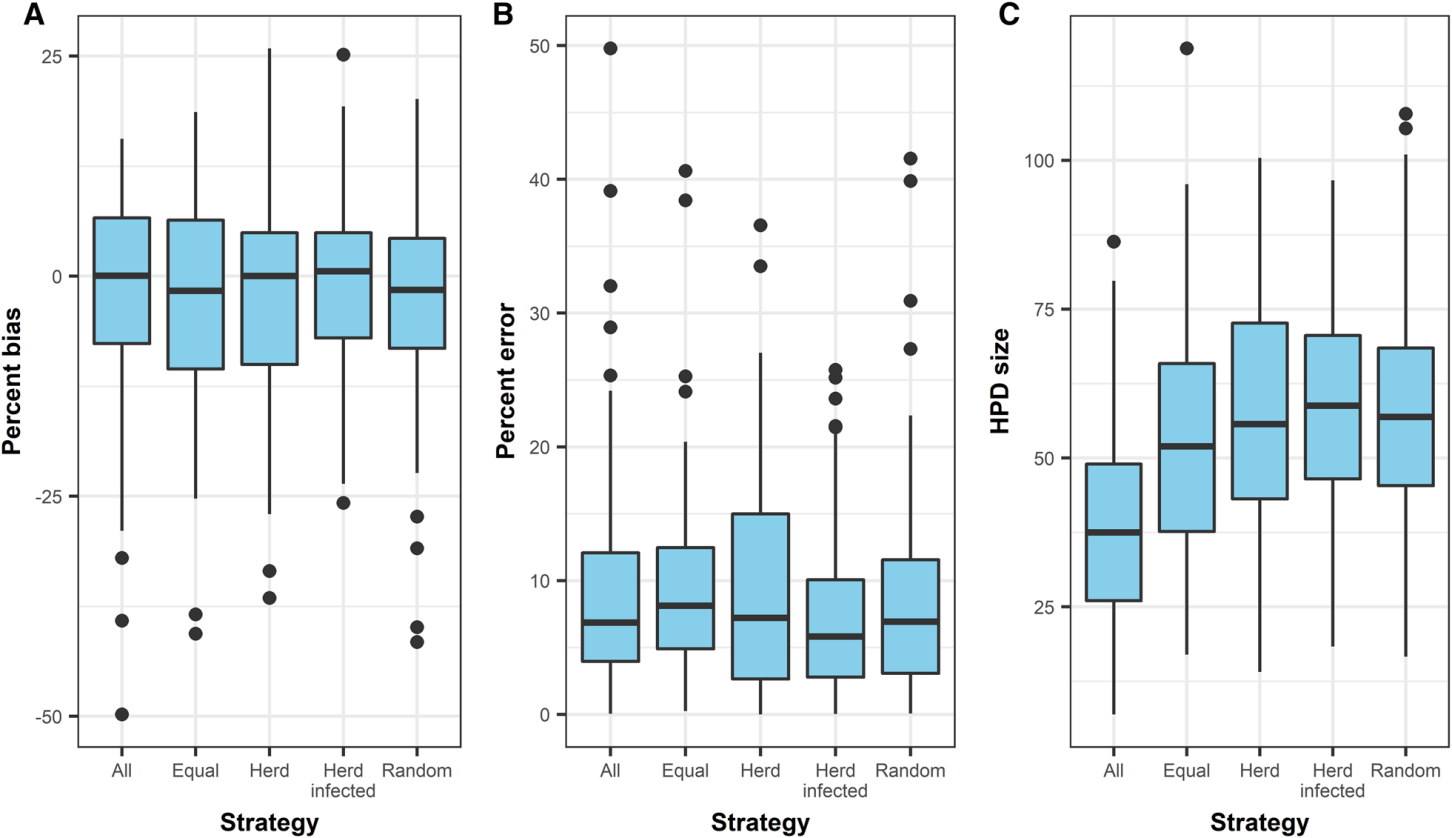
**Comparisons of accuracy and precision of phylodynamic analyses in different subsampling strategies.**

Figure 6 and Table 1 show the distributions of percent bias, percent error, and HPD size from each subsampling strategy. For percent bias and percent error, there was no evidence that distributions from any subsampling strategies were different from the distribution from the “All” strategy, in which all available genetic samples were used (Kolmogorov–Smirnov test, all *p* values > 0.3). On 84 occasions of 100 simulated epidemics, at least one of four subsampling strategies provided a smaller percent error compared to the “All” strategy. On the other hand, there was a strong evidence that the distribution of HPD size from the “All” strategy was different from distributions from any other subsampling strategies (all *p*-values < 0.00002).

**Table 1:** **Performance of subsampling strategies and the scenario in which all available genetic samples were used (“All” strategy) in the phylodynamic analysis**

Strategy	Percent bias	Percent error	HPD size	Coverage (%)	Convergence (%)
All	0.05 (−7.7, 6.6)	6.87 (0.23, 30.6)	37.5 (10.3, 77.5)	91	59
Equal	−1.66 (−10.5, 6.4)	8.13 (0.6, 24.7)	52.0 (20.0, 93.3)	98	93
Random	−1.54 (−8.2, 4.3)	6.94 (0.28, 29.2)	56.9 (19.5, 100.3)	99	92
Herd infected	0.58 (−7.0, 4.9)	5.84 (0.34, 22.6)	58.8 (25.4, 88.5)	99	95
Herd	0.02 (−10.0, 4.9)	7.22 (0.21, 26.5)	55.7 (24.2, 91.8)	99	93

Coverage indicates the proportion of 95% HPD that contained the true value of the last sampling time out of 100 iterations. Convergence metric is a rudimentary measure to indicate how many iterations failed to converge within 50 000 000 MCMC iterations. A longer chain was run if a convergence did not occur to obtain TMRCA estimates.

The 95% HPD of TMRCA estimates in any of the subsampling strategies included the “true” value nearly 100 times, whereas those from the “All” failed to include “true” value 9 out of 100 times. A convergence occurred within 50 000 000 iterations in more than 90 occasions out of 100 for each of subsampling strategy. However, a convergence within this iteration only occurred 59 times for the “All” strategy, and hence it was necessary to run a longer Markov chain to achieve convergences.

### Comparison of performance between subsampling strategies

We finally carried out a rudimentary evaluation on whether one subsampling strategy provides a smaller percent error than any other strategies using Wilcoxon paired tests on all possible pairwise combinations of strategies. There was no evidence to support one strategy performs better than others except between the “Equal” and the “Herd infected” (unadjusted *p*-value = 0.016) and between the “Herd infected” and the “Herd” (unadjusted *p*-value = 0.039).

## Discussion

To the best of our knowledge, this is the first study to quantify the degree of bias in the popular BEAST analysis to estimate the time to the most recent common ancestor (TMRCA) in the presence of super-spreaders using different population sampling strategies.

Although the median percent error in TMRCA estimates over 100 simulated outbreaks was relatively small when all available genetic samples were analysed in the Extended Bayesian Coalescent Skyline plot (EBSP) and the Birth–death skyline plot (BDSKY) model framework, substantial biases were observed in several occasions. We tested how the presence of super-spreaders influenced the accuracy of phylodynamic inferences. While tested variables related to super-spreader did not show any associations with the accuracy of EBSP model, that of BDSKY model was more deteriorated as a larger proportion of infected farms was generated by a super-spreader. Exactly the same trend was observed for the precision of inferences (i.e. HPD size); the presence of a dominant super-spreader deteriorated the HPD size of BDSKY but not of EBSP. By contrast, the epidemic duration seemed to be an important factor for inferences using EBSP model: the shorter an epidemic, the less accurate and precise an estimate. The difference in the mechanism of inducing bias between EBSP and BDSKY might be partially due to the difference in how the coalescent and birth–death models treat sampling times and indirectly create a tree height prior as discussed in [25]. For the coalescent model, the presence of super-spreaders in the epidemic may directly bias the coalescent rate (and the effective population size: EPS) in a given time interval. By contrast, this may directly bias the birth and death rate in a given time interval in the birth–death model. We therefore speculate the degree of bias may be associated with the number of dimensions set for EPS and effective reproduction number parameter, respectively. We only tested the static measure of super-spreaders (i.e. proportion of secondary cases generated by a super-spreader). Future studies need to study how the temporality of super-spreaders influence phylodynamic inferences (e.g. do super-spreaders exist in the early or late phase of an epidemic?).

While the accuracy of the least-squares dating (LSD) was correlated with the degree of the temporal signal contained in the sequences, this was not the case for EBSP and BDSKY models. This is reasonable given that the LSD method requires a stronger temporal signal, and that we used very narrow prior distributions for the clock rate (which contained the true value), which enabled the models to tease apart tree height estimates from clock rate estimates [25]. Our finding suggests that bias in TMRCA estimates can still occur even when a moderate temporal signal exists and a narrow prior can be set on the clock rate. We also assumed “true” substitution model is known and used Jukes–Cantor model in the BEAST analysis. In real circumstances however, it is necessary to perform a substitution model selection or model averaging approach [60, 61]. This process was skipped to exclude potential bias due to misspecifying a substitution model. In fact, using jModelTest [62], Jukes–Cantor model was chosen as most appropriate based on BIC in 46% of our simulated outbreaks (although the difference in BIC between the best model and Jukes–Cantor model was less than 6 in majority of simulated dataset, suggesting the evidence that favours other models than Jukes–Cantor was weak).

The overall percent error distributions from EBSP were similar irrespective of whether all available genetic samples or a subset of samples were used. Nevertheless, at least one of the subsampling strategies performed better than the “All” strategy in at least 84% of simulated epidemics. On the other hand, the “All” strategy provided substantially more precise estimates (narrower credible intervals), which is not surprising because an inclusion of more samples can reduce a random error. However, narrower HPDs do not necessarily mean better because the point estimates are systematically biased and hence narrower HPDs may fail to include “true” value as we showed in this analysis. Moreover, it was necessary to run a longer Markov chain to achieve a convergence when all samples were used. Taken together, this suggests that it is not advisable to use all available genetic samples when the objective is to estimate the TMRCA for a disease that spreads over a complex livestock network, although this recommendation may be contrary to what has been believed in the past veterinary studies [63, 64]. A large-scale simulation study carried out by the PANGEA-consortium, however, indeed reported that low sampling coverage does not contribute to substantial errors in various phylodynamic inferences in their dataset mimicking HIV genetic sequences sampled before and after the intervention in sub-Saharan Africa [65].

A previous modelling study suggested that the “Equal” subsampling strategy—uniform probability sampling with respect to time and geographical areas—may be a preferred subsampling strategy [32]. In our study, however, there was no evidence that the “Equal” strategy outperformed other subsampling strategies. The differences in findings may be partially due to the difference in the disease transmission systems used in these two studies. The former study assumed that a disease spreads through a homogeneous mixing manner in each of six population structures with a constant transmission between each structure. In contrast, we assumed that disease spreads through livestock movements, which can occur independently from the farm geographical locations (i.e. long distance trade movements). For livestock diseases that spread through direct contacts, geographical locations are therefore not entirely representative of the true population structure. This raises an important question as to how we can define a population structure for such diseases. Answering this question will allow us to use tree generating models that have been developed for structural populations [13–16, 33]. It would be interesting to test whether a structured model can reduce bias by treating samples from super-spreader farm and farms infected by the super-spreader (or farms belonging to the same network community as the super-spreader) as one population structure.

Future studies need to identify more precise inclusion criteria for pathogen sequences in phylodynamic analyses. Although there was some evidence that the “Herd infected” strategy may provide a smaller percent error than some of other subsampling strategies, we should not interpret that this strategy systematically performs better than others because our simulation scenarios were limited. Rather, this finding suggests that the decision of which sequences to include has a substantial impact on the estimate and that this decision may need to be tailored to each epidemic. We speculate that the performance of subsampling may be associated with the shape of transmission tree, size and number of clustering, and which nodes to sample from a given tree shape. Future studies are warranted to investigate this association using a wide range of statistics describing tree topologies and branch lengths such as those used in the recent study [66]. While sampling based on a transmission tree topology may not be realistic given that the true transmission trees are almost always unknown, it is possible to sample based on an estimated phylogenetic tree.

This study suggested that a farm that exports animals to ≥ 19 farms per year can act as a super-spreader and the presence of these farms in transmission chains may lead to a biased phylodynamic inference. However, this result should not be used as a general guideline. Several key assumptions made in this study, such as transmissions only occurring through direct contacts, may be not realistic or applicable to other situations. We also selected a subset of farms as a seed and this may have limited the disease spread patterns and hence the potential variations in phylodynamic inferences. These limitations can be, however, easily overcome by applying our framework using livestock movement patterns in other countries and more complex disease scenarios such as when mutations rates differ in each disease status. Given our framework simultaneously simulates a disease spread and mutations, rather than simulating mutations post hoc on transmission trees created from disease models, it is straightforward to account for interactions between an epidemiological and evolutionary process. Ideally, a similar study to the one conducted by the PANGEA-consortium [65] should be carried out to evaluate the degree of bias in the phylodynamic estimates for livestock and zoonotic diseases to identify methodological improvements required to provide robust inferences.

In conclusion, we quantified the bias in the estimate of the time to most recent common ancestor (TMRCA) arising from applying two popular tree generating models (Extended Bayesian Coalescent skyline plot: EBSP, and Birth–death skyline plot: BDSKY) when a disease spreads through a super-spreader within a complex contact structure. Although the homogeneous mixing assumption made in these models is likely to be violated, the degree of bias in TMRCA estimates was relatively small in the majority of simulated outbreaks provided that an accurate and precise prior for molecular clock rate was used. Nevertheless, substantial bias was observed in several epidemics in both EBSP and BDSKY models. The degree of bias was influenced by different factors in each model and this degree was irrespective of the strength of the temporal signal contained in the genetic sequences (again, provided that narrow prior on the clock rate is used). When compared to the performance of using all available samples, subsampling of sequences provided similar or more accurate estimates in the majority of simulated epidemics with less computational time being required. Using all available genetic sequences to estimate TMRCA is therefore not recommended and various subsampling strategies should be applied to examine how estimates are sensitive to inclusions of different samples.

## Supplementary information

**Additional file 1. Description of livestock movement data used for a disease simulation.** This file provides various demographic statistics on the movement data used.

**Additional file 2. Degree distributions for each age group.** Movements were aggregated over a year separately for each age group from 2000 to 2010. Outdegree was defined as the number of farms a given farm sent at least one animal in a year for a given age category. Indegree was defined as the number of farms a given farm received at least one animal in a year for a given age category. Outdegree and indegree distributions for calf, heifer, adult, and all categories combined for 2000 to 2002 are shown.

**Additional file 3. Network statistics.** Distributions of farm-level network statistics (A) Density, (B) Cluster coefficient, (C) Connectedness, and (D) Reciprocity, calculated for a yearly-aggregated directed network for each age category from 2000 to 2010.

**Additional file 4. Statistics describing the fit of power law.** Fit of power law to a yearly-aggregated outdegree (A, B, and C) and indegree (D, E, and F) distributions for 2000 to 2010. (A) and (D) show the distributions of exponent of power law, (B) and show good of fitness, and (C) and (F) show bootstrap p-value which indicate the likeliness of a distribution being drawn from power law. Details of methods can be found in Additional file 5.

**Additional file 5. Methods of fitting power law**. Detailed methods and results of fitting power law to the in- and outdegree of livestock movement data from 2000 to 2010.

**Additional file 6. Simulation parameter values**. This file provides parameter values used in the disease simulation.

**Additional file 7. Exemplar transmission trees.** Exemplar transmission trees with different Sackin index scores. Each circle represents an infected farm. Red circles represent farms which did not infect any other farms (i.e. leaves). Blue circles represent index farms. Tree (A) is highly imbalanced with a normalised Sackin index −6.2 (substantially different from 0). Tree (B) is relatively balanced with a normalised Sackin index −2.3 (closer to 0). While tree (A) has many leaves with relatively short path lengths from the index farm, tree (B) has much fewer leaves.

**Additional file 8. The BEAST setting for Birth–death Skyline plot model.** This file provides a detailed explanation on how BDSKY model was set.

**Additional file 9. Distribution of the maximum number of secondary cases (R) over 100 simulations.** This histogram shows the distribution of the largest number of farms that were infected by a single farm.

**Additional file 10. Distributions of number of secondary cases (R) in each epidemic over 100 simulations**. This figure overlays 100 distributions of the number of farms that were infected by a single farm.

**Additional file 11. Farm characteristics of super-spreaders identified in 100 simulations.** This file provides a detailed explanation on super-spreader farms identified in this study.

**Additional file 12. Degree distributions for super-spreaders**. Distributions of outdegree (A, B) and indegree (C, D) for 18 farms that were defined as super-spreaders. These farms infected equal to or larger than 20 farms in at least 1 outbreak. The x-axis represents each farm (A to R), each showing statistics calculated for calf (red), heifer (green), and adult (blue) movement networks aggregated over a year. Boxplots summarise statistics for 5 years (2000 to 2004). The dashed red lines indicate the median value for each statistics, calculated irrespective of age categories (i.e. movement networks were aggregated over a year for all age groups). These median values were calculated only including farms that had none-zero values for each statistics.

**Additional file 13. Demographics of supers-spreaders**. Distributions of the number of animals in each age category in Year 2000 across 18 super-spreader farms. The red dashed lines indicate the median values calculated using all farms existed in Year 2000 in the data.

**Additional file 14. Descriptive statistics of simulation results.** Descriptive statistics of results obtained from individual-based disease simulation models over 100 simulated outbreaks. The red dashed lines represent the median values for each statistic.

**Additional file 15. Association between the temporal signal strength and the percent error.** Scatter plots showing no association between R squared value and (A) the percent error of EBSP and (B) the percent error of BDSKY model. Pearson’s correlation coefficients were 0.04 (*p*-value = 0.69) and -0.0007 (*p*-value = 0.99) for EBSP and BDSKY, respectively.

**Additional file 16. Correlation of the accuracy and precision between three models.** Scatter plots comparing the accuracy and precision statistics between EBSP, BDSKY, and LSD model. (A) to (C): the percent bias, (D) to (F): the percent error, and (G): the HPD size between EBSP and BDSKY. Pearson’s correlation coefficients and p-values are as follows: (A): 0.72 (*p*-value < 0.0001), (B): −0.024 (*p*-value = 0.8), (C): −0.019 (*p*-value = 0.8), (D): 0.52 (*p*-value < 0.0001), (E): 0.04 (*p*-value = 0.67), (F): −0.02 (*p*-value = 0.8), and (G) 0.68 (*p*-value < 0.0001). The blue lines represent x = y.

**Additional file 17. Other results of Birth–death Skyline model**. Estimates on the become uninfectious rate (A) and the sampling proportion after the first sequence being collected (B) from BDSKY model. The red lines in (A) indicate the lower and upper limit of the same parameter used in the disease simulation.

**Additional file 18. Univariable associations between each epidemic characteristic and the percent error of the EBSP model**. A table describing the result of the univariable linear regression model

**Additional file 19. Univariable associations between each epidemic characteristic and the percent error of the BDSKY model.** A table describing the result of the univariable linear regression model.

**Additional file 20. Univariable associations between each epidemic characteristic and the HPD size of the EBSP model**. A table describing the result of the univariable linear regression model.

**Additional file 21. Univariable associations between each epidemic characteristic and the HPD size of the BDSKY model**. A table describing the result of the univariable linear regression model.

**Additional file 22. Results of the multivariable models.** A table describing the results of the final multivariable linear regression model for each statistic.

## Data Availability

The datasets generated during the current study are available in the first author’s GitHub repository [52].
