# Supplementary material for: Assessing biases in phylodynamic inferences in the presence of super-spreaders
Source: Vet Res. 2019 Sep 27;50:74. doi: 10.1186/s13567-019-0692-5 (PMC6764146; doi:10.1186/s13567-019-0692-5)
Supplement: Supplementary file 1 — Additional file 1. Description of livestock movement data used for a disease simulation. This file provides various demographic statistics on the movement data used. [file 13567_2019_692_MOESM1_ESM.docx]

**Additional file 1 Description of livestock movement data used for a disease simulation.**

| Variable | Values |
| --- | --- |
| The number of animals present in the database as of 1^st^ July 2000 | 3 624 420 |
| The total number of animals recorded in the study period (1^st^ July 2000 to 31^st^ June 2000) | 16 534 951 |
| The total number of distinct herds recorded in the study period | 45 965 |
| The number of parturition records in the study period | 9 667 100 |
| The number of culling records in the study period | 6 165 850 |
| The number of movement records in the study period | 8 841 850 |
| The number of movement records that were suspected to be missing^1^ | 18 717 |
| The number of distinct animals that had moved between farms | 5 487 015 (100%) |
| The number of distinct calves that had moved | 1 480 616 (16.7%) |
| The number of distinct heifers that had moved | 2 000 401 (22.6%) |
| The number of distinct adults that had moved | 5 379 550 (60.7%) |
| The number of between-farm moves made by these animals | 8 860 567 |
| The number of distinct source farms | 29 223 |
| The number of distinct destination farms | 25 613 |

^1^ Missing data was suspected because the destination herd of a movement does not match to the source herd of an immediate next movement for the same animal. On these occasions, we simply added a movement from the destination herd to the source herd, sampling a random date between these two movements, assuming there is only one missing record in each occasion.
