# Supplementary figures and images for: Assessing biases in phylodynamic inferences in the presence of super-spreaders
Source: Vet Res. 2019 Sep 27;50:74. doi: 10.1186/s13567-019-0692-5 (PMC6764146; doi:10.1186/s13567-019-0692-5)

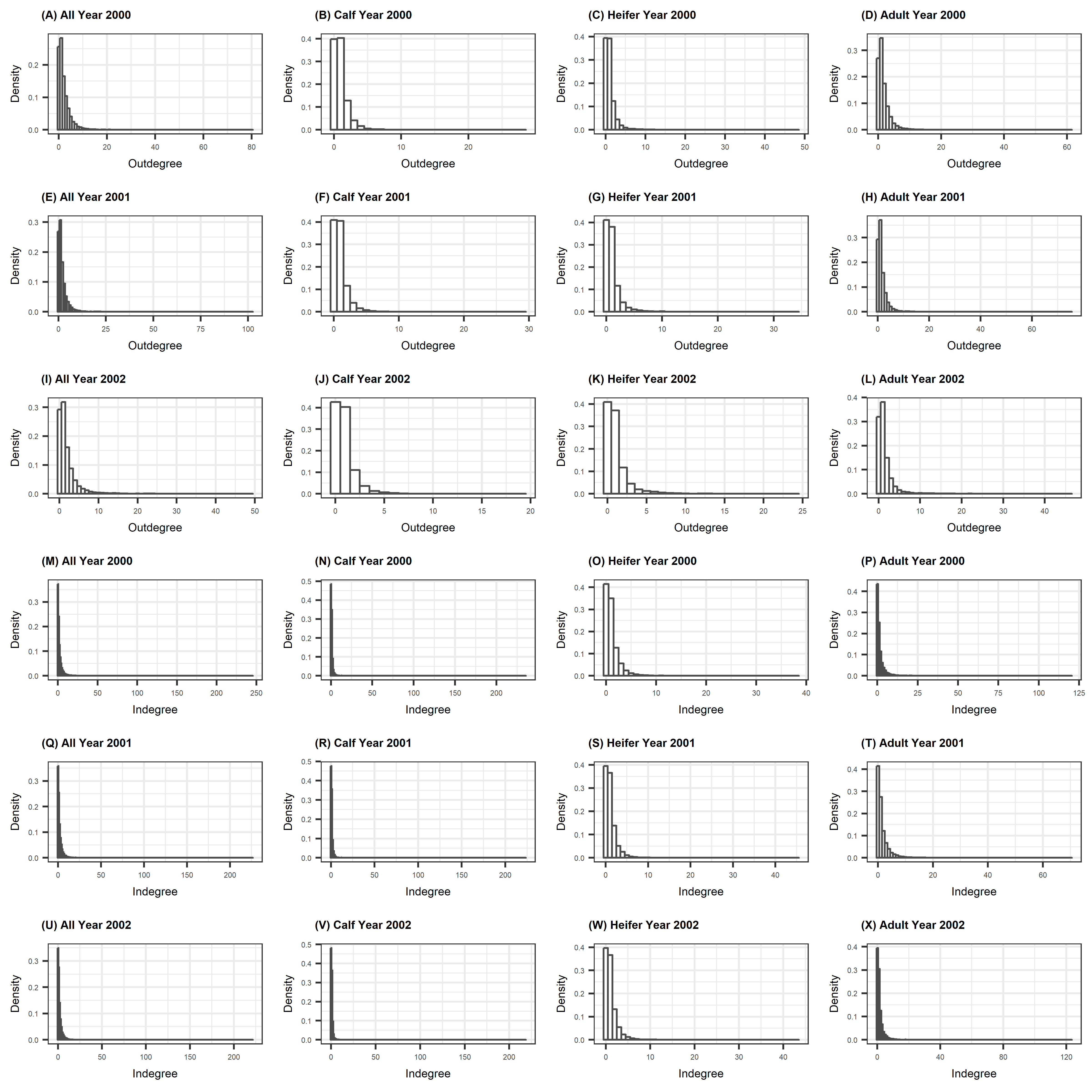

Supplement: Supplementary file 2 — Additional file 2. Degree distributions for each age group. Movements were aggregated over a year separately for each age group from 2000 to 2010. Outdegree was defined as the number of farms a given farm sent at least one animal in a year for a given age category. Indegree was defined as the number of farms a given farm received at least one animal in a year for a given age category. Outdegree and indegree distributions for calf, heifer, adult, and all categories combined for 2000 to 2002 are shown. [file 13567_2019_692_MOESM2_ESM.tiff]

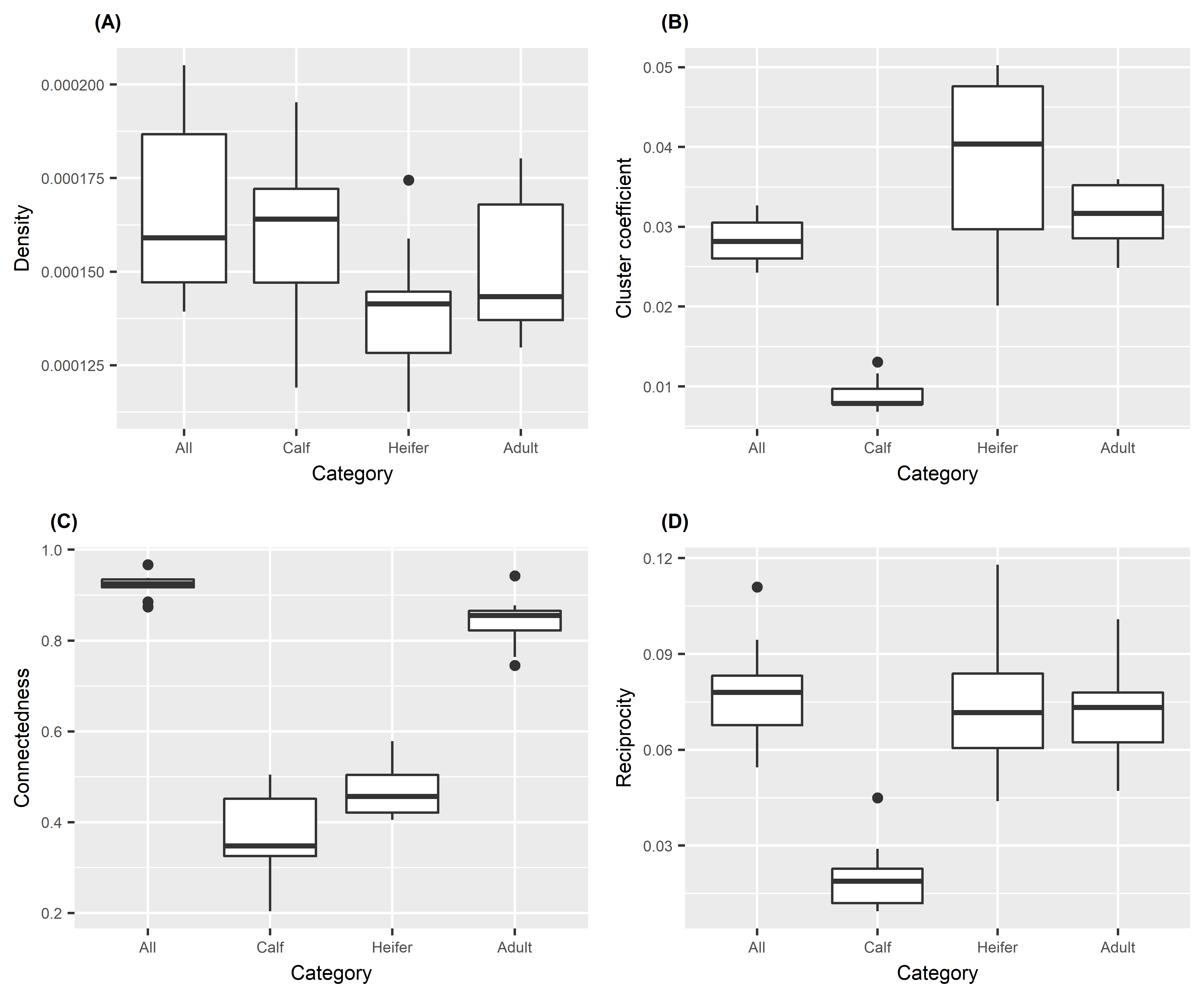

Supplement: Supplementary file 3 — Additional file 3. Network statistics. Distributions of farm-level network statistics (A) Density, (B) Cluster coefficient, (C) Connectedness, and (D) Reciprocity, calculated for a yearly-aggregated directed network for each age category from 2000 to 2010. [file 13567_2019_692_MOESM3_ESM.tiff]

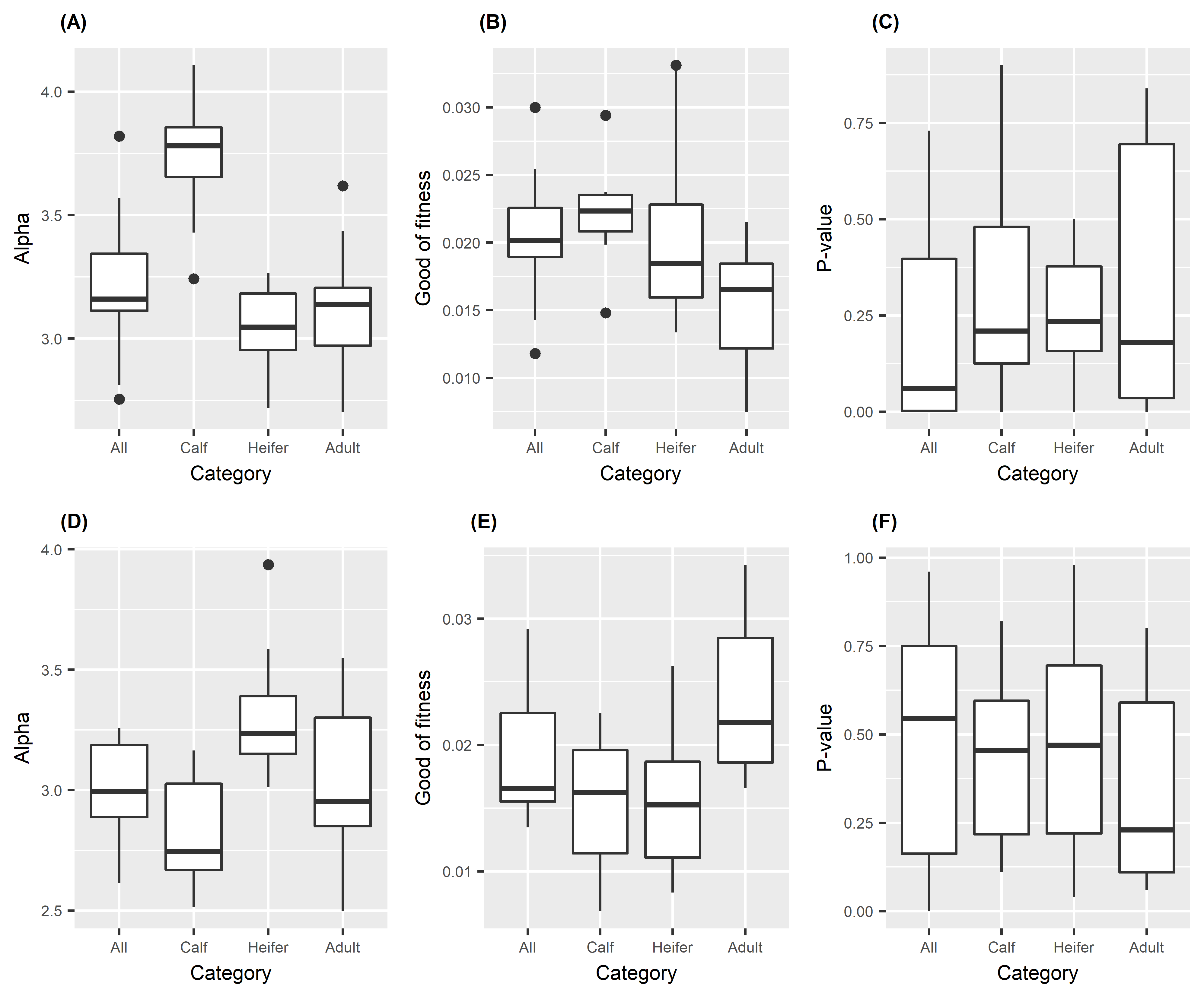

Supplement: Supplementary file 4 — Additional file 4. Statistics describing the fit of power law. Fit of power law to a yearly-aggregated outdegree (A, B, and C) and indegree (D, E, and F) distributions for 2000 to 2010. (A) and (D) show the distributions of exponent of power law, (B) and show good of fitness, and (C) and (F) show bootstrap p-value which indicate the likeliness of a distribution being drawn from power law. Details of methods can be found in Additional file 5. [file 13567_2019_692_MOESM4_ESM.tiff]

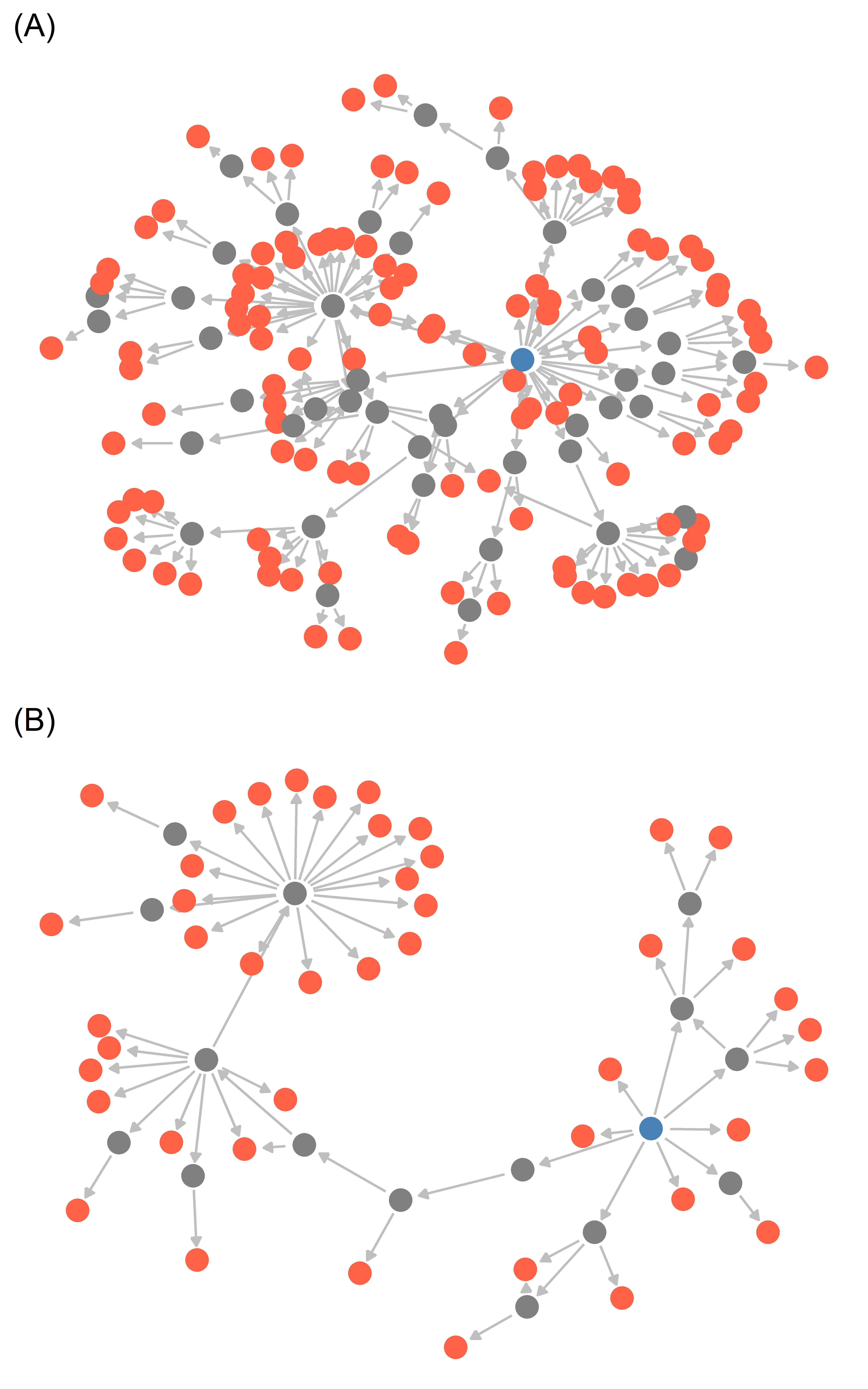

Supplement: Supplementary file 7 — Additional file 7. Exemplar transmission trees. Exemplar transmission trees with different Sackin index scores. Each circle represents an infected farm. Red circles represent farms which did not infect any other farms (i.e. leaves). Blue circles represent index farms. Tree (A) is highly imbalanced with a normalised Sackin index −6.2 (substantially different from 0). Tree (B) is relatively balanced with a normalised Sackin index −2.3 (closer to 0). While tree (A) has many leaves with relatively short path lengths from the index farm, tree (B) has much fewer leaves. [file 13567_2019_692_MOESM7_ESM.tiff]

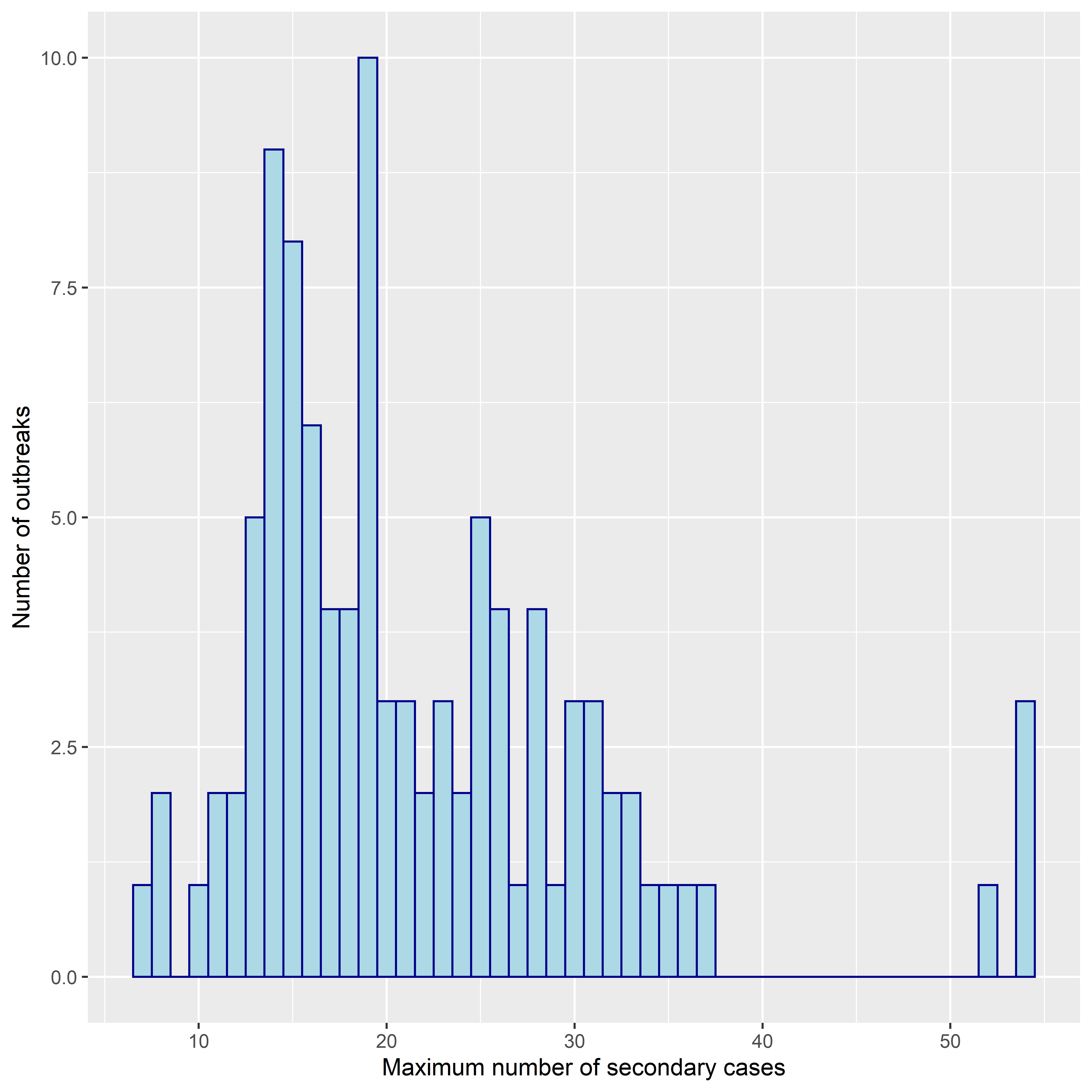

Supplement: Supplementary file 9 — Additional file 9. Distribution of the maximum number of secondary cases (R) over 100 simulations. This histogram shows the distribution of the largest number of farms that were infected by a single farm. [file 13567_2019_692_MOESM9_ESM.tiff]

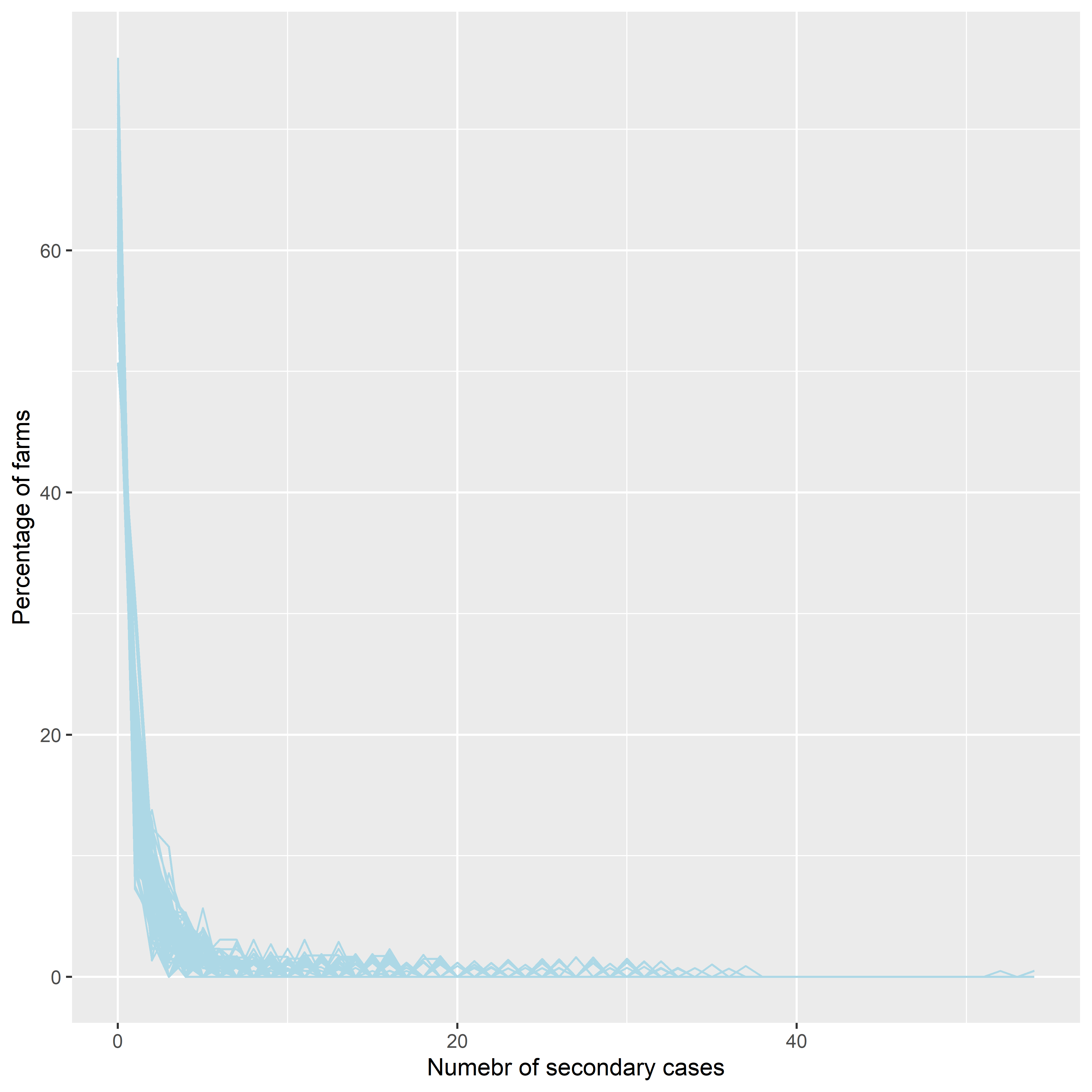

Supplement: Supplementary file 10 — Additional file 10. Distributions of number of secondary cases (R) in each epidemic over 100 simulations. This figure overlays 100 distributions of the number of farms that were infected by a single farm. [file 13567_2019_692_MOESM10_ESM.tiff]

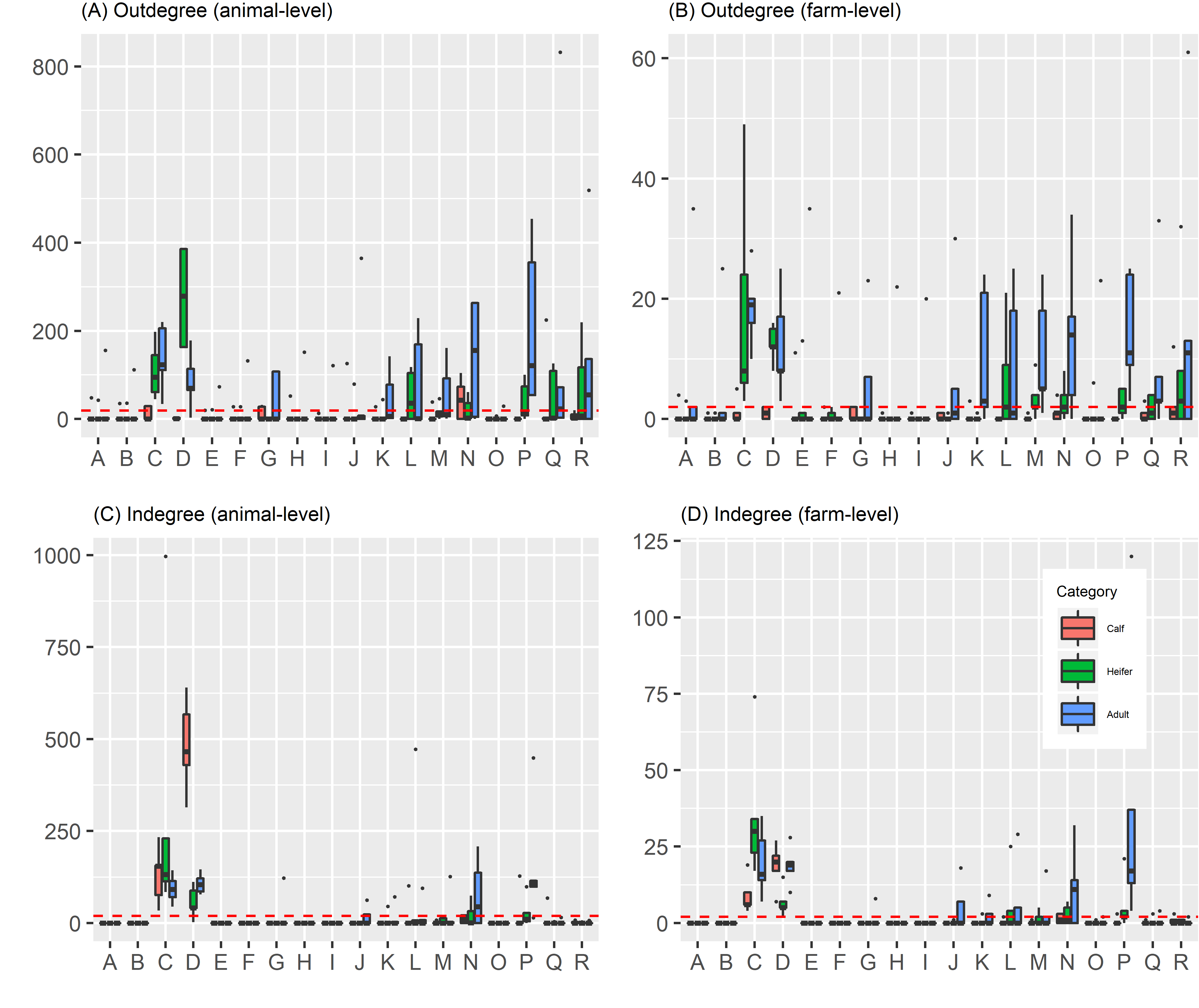

Supplement: Supplementary file 12 — Additional file 12. Degree distributions for super-spreaders. Distributions of outdegree (A, B) and indegree (C, D) for 18 farms that were defined as super-spreaders. These farms infected equal to or larger than 20 farms in at least 1 outbreak. The x-axis represents each farm (A to R), each showing statistics calculated for calf (red), heifer (green), and adult (blue) movement networks aggregated over a year. Boxplots summarise statistics for 5 years (2000 to 2004). The dashed red lines indicate the median value for each statistics, calculated irrespective of age categories (i.e. movement networks were aggregated over a year for all age groups). These median values were calculated only including farms that had none-zero values for each statistics. [file 13567_2019_692_MOESM12_ESM.tiff]

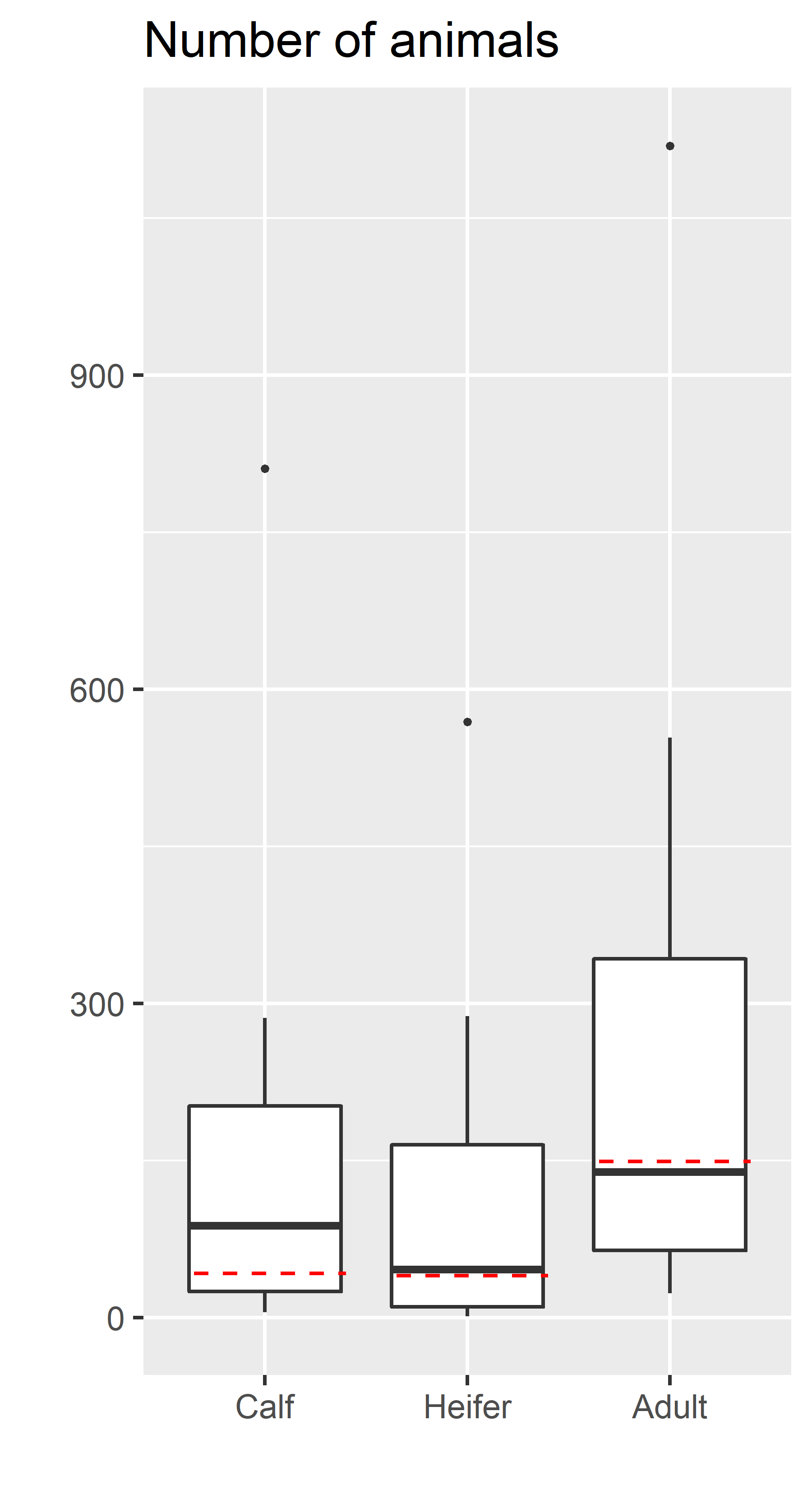

Supplement: Supplementary file 13 — Additional file 13. Demographics of supers-spreaders. Distributions of the number of animals in each age category in Year 2000 across 18 super-spreader farms. The red dashed lines indicate the median values calculated using all farms existed in Year 2000 in the data. [file 13567_2019_692_MOESM13_ESM.tiff]

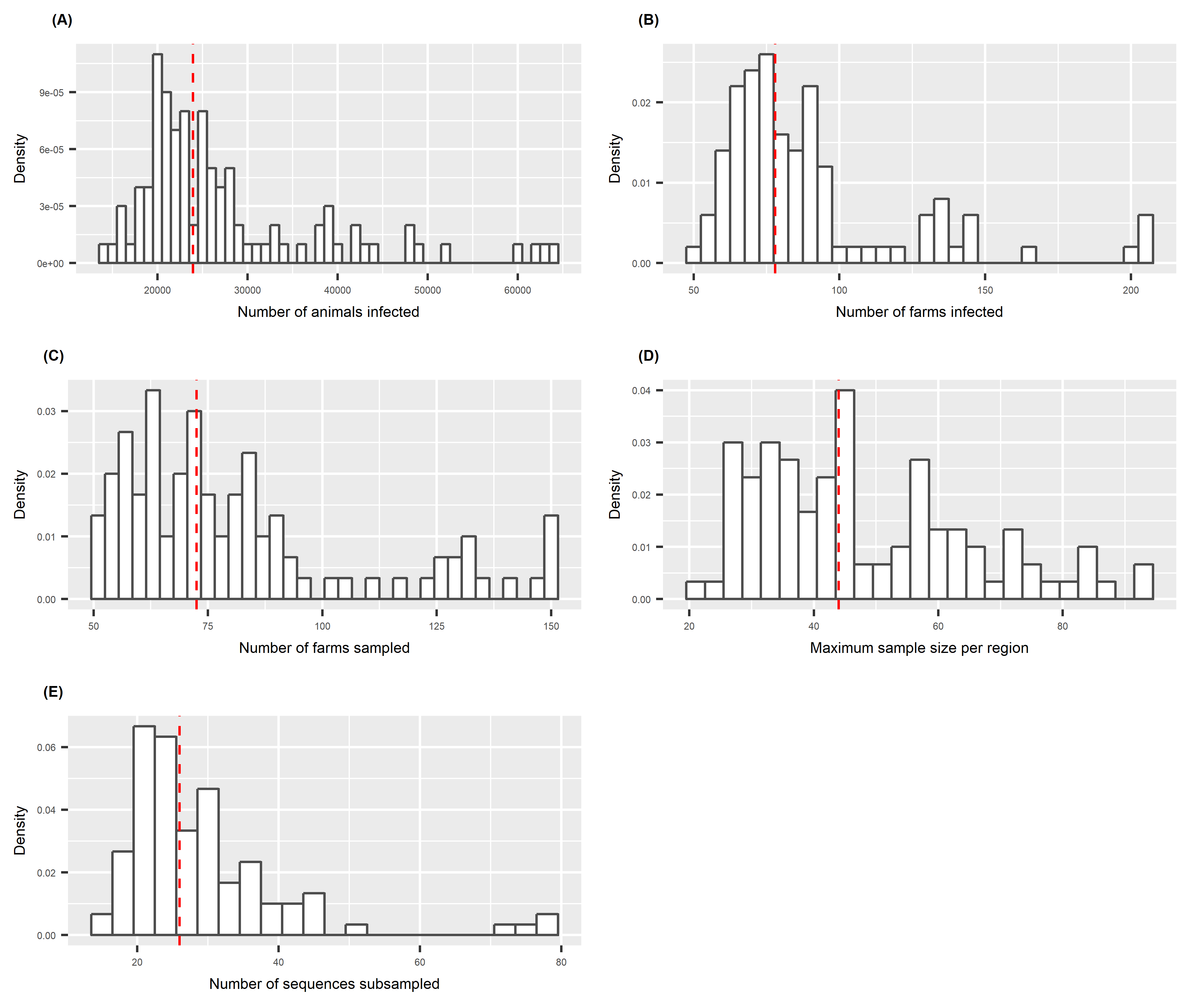

Supplement: Supplementary file 14 — Additional file 14. Descriptive statistics of simulation results. Descriptive statistics of results obtained from individual-based disease simulation models over 100 simulated outbreaks. The red dashed lines represent the median values for each statistic. [file 13567_2019_692_MOESM14_ESM.tiff]

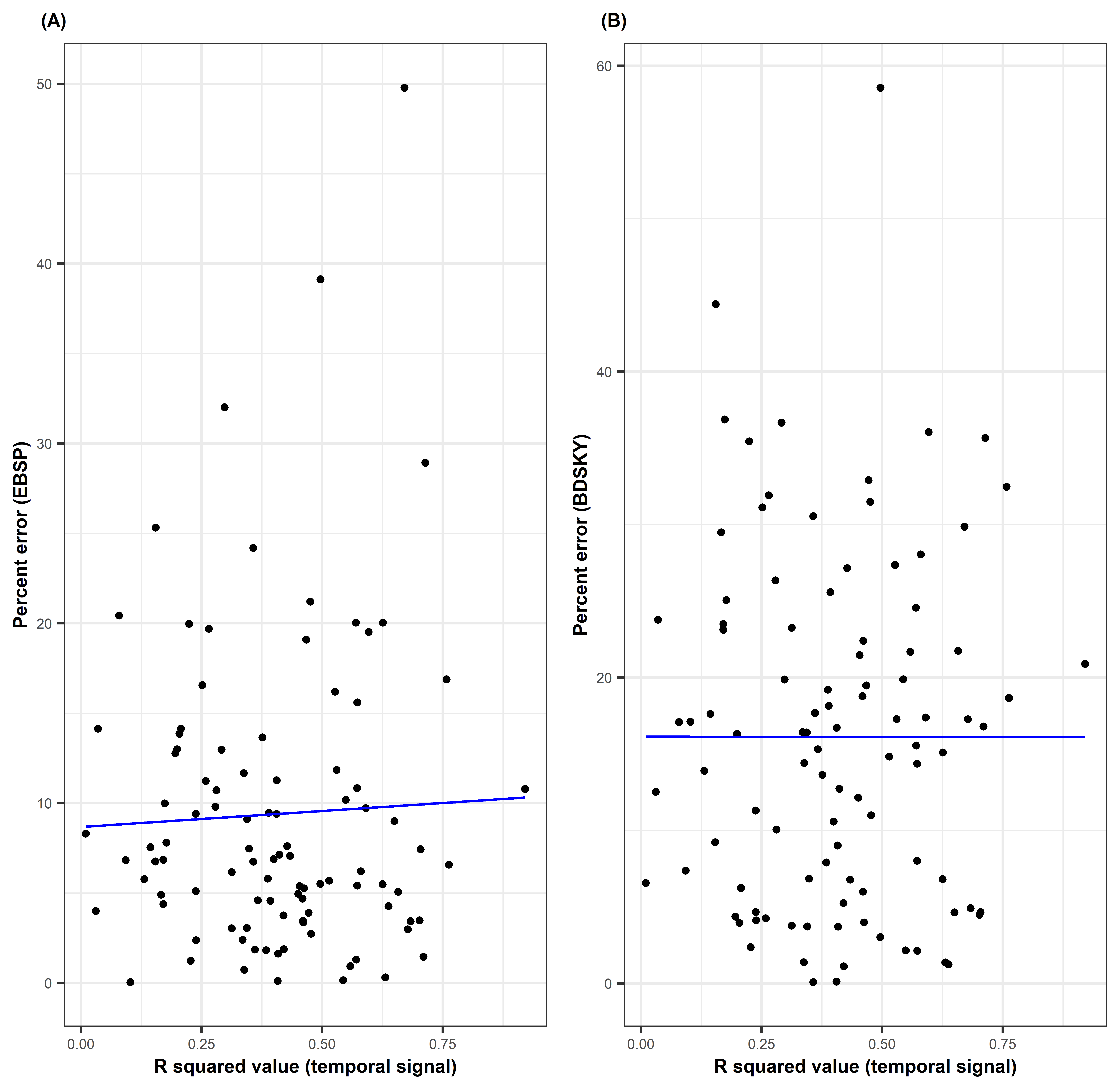

Supplement: Supplementary file 15 — Additional file 15. Association between the temporal signal strength and the percent error. Scatter plots showing no association between R squared value and (A) the percent error of EBSP and (B) the percent error of BDSKY model. Pearson’s correlation coefficients were 0.04 (p-value = 0.69) and -0.0007 (p-value = 0.99) for EBSP and BDSKY, respectively. [file 13567_2019_692_MOESM15_ESM.tiff]

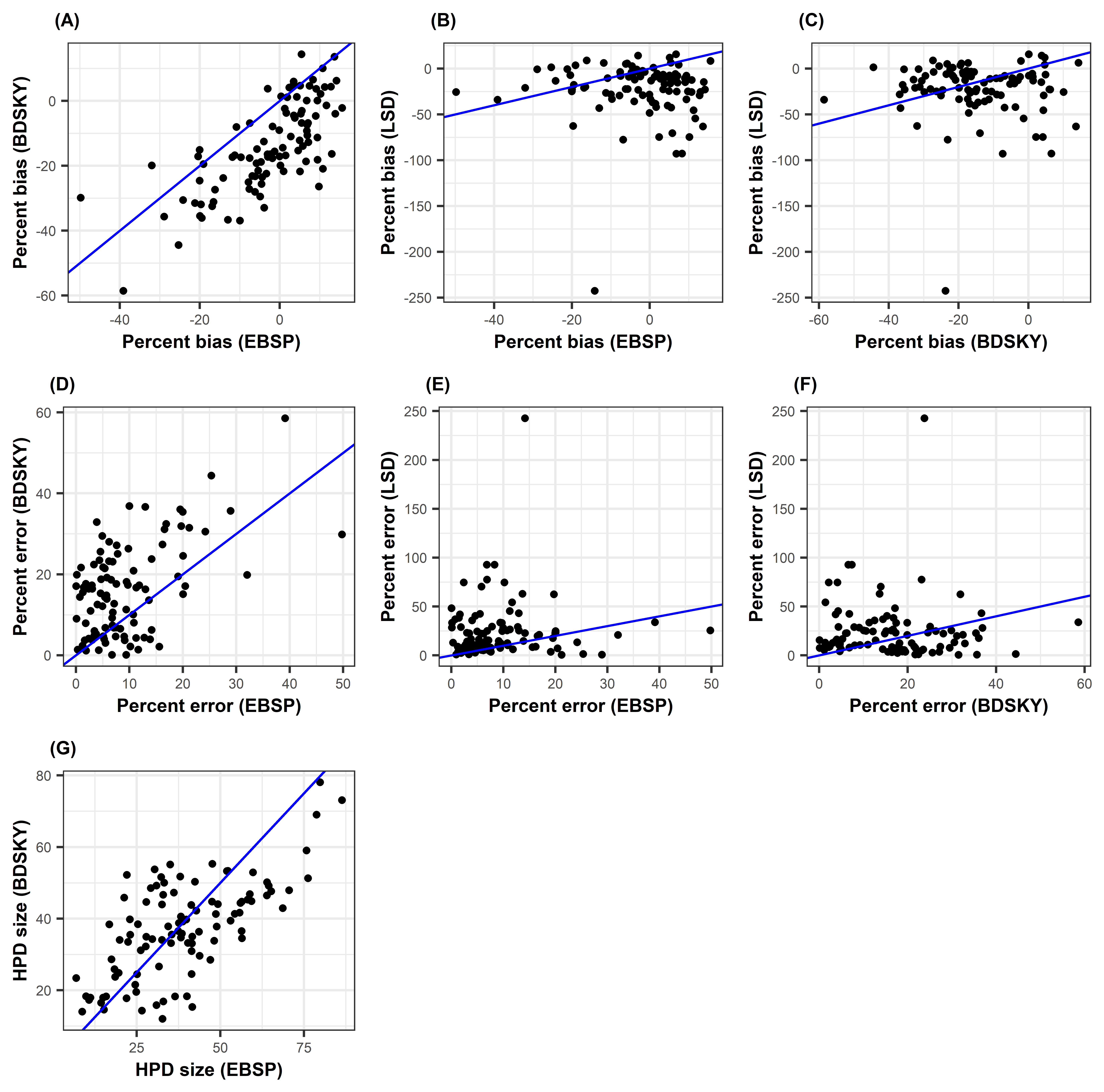

Supplement: Supplementary file 16 — Additional file 16. Correlation of the accuracy and precision between three models. Scatter plots comparing the accuracy and precision statistics between EBSP, BDSKY, and LSD model. (A) to (C): the percent bias, (D) to (F): the percent error, and (G): the HPD size between EBSP and BDSKY. Pearson’s correlation coefficients and p-values are as follows: (A): 0.72 (p-value < 0.0001), (B): −0.024 (p-value = 0.8), (C): −0.019 (p-value = 0.8), (D): 0.52 (p-value < 0.0001), (E): 0.04 (p-value = 0.67), (F): −0.02 (p-value = 0.8), and (G) 0.68 (p-value < 0.0001). The blue lines represent x = y. [file 13567_2019_692_MOESM16_ESM.tiff]

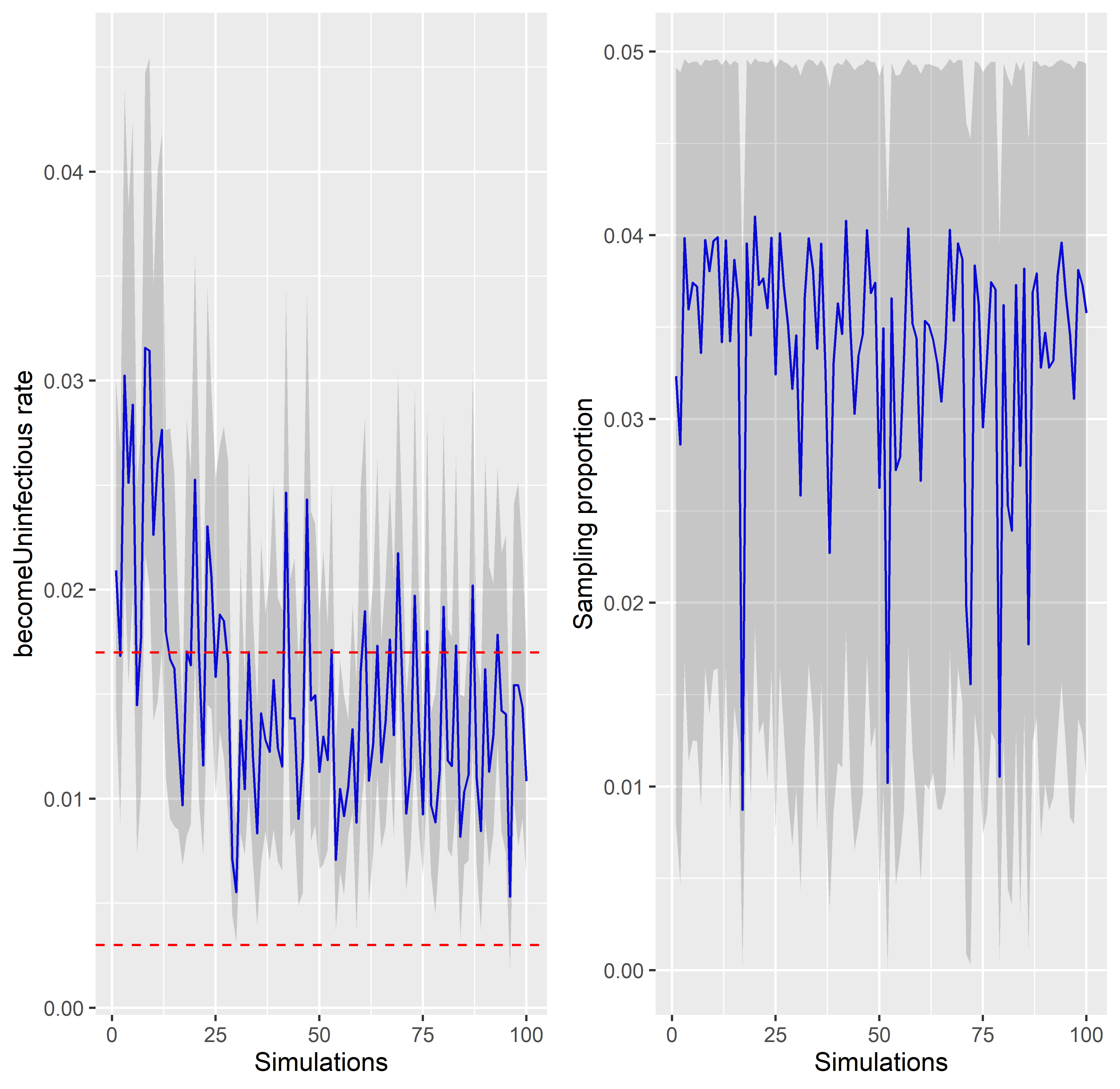

Supplement: Supplementary file 17 — Additional file 17. Other results of Birth–death Skyline model. Estimates on the become uninfectious rate (A) and the sampling proportion after the first sequence being collected (B) from BDSKY model. The red lines in (A) indicate the lower and upper limit of the same parameter used in the disease simulation. [file 13567_2019_692_MOESM17_ESM.tiff]
