# Supplementary material for: Assessing biases in phylodynamic inferences in the presence of super-spreaders
Source: Vet Res. 2019 Sep 27;50:74. doi: 10.1186/s13567-019-0692-5 (PMC6764146; doi:10.1186/s13567-019-0692-5)
Supplement: Supplementary file 5 — Additional file 5. Methods of fitting power law. Detailed methods and results of fitting power law to the in- and outdegree of livestock movement data from 2000 to 2010. [file 13567_2019_692_MOESM5_ESM.docx]

**Additional file 5** **Fitting outdegree and indegree of the network into a distribution**

Method

Individual livestock movement records of each year from 1^st^ July 2000 to 30^th^ June 2010 were aggregated over a farm level, which was carried out for calf, heifer, adult, and all age groups, respectively. Directed graphs were then created based on each movement network, which provided distributions for outdegree (i.e. to how many farms each farm sent at least one animal) and indegree (i.e. from how many farms each farm received at least one animal). Hence, there were 80 distributions (out/in degree, 4 age groups, 10 years). For each of distribution, we fitted a power law, exponential, log normal, and Poisson distribution using R package poweRlaw. A 100 times bootstrap procedure was performed to provide p-value that indicates how likely we get the obtained Kolmogorov–Smirnov statistics when the data was drawn from a power law distribution (hence *p*-value >= 0.05 was deemed that it is likely that the data was drawn from a power law distribution). The Vuong’s test was then carried out to examine whether either exponential, log normal or Poisson distribution fits better than a power law distribution.

Result

Out of 40 outdegree distributions, it is unlikely that the data was drawn from a power law distribution on 12 occasions: 5 for all age groups, 2 for calf, 2 for heifer, and 3 for adult age group. Out of these 12 distributions, a log normal distribution showed a better fit for 4 distributions. For 1 distribution that was likely to be from a power law distribution, a log normal distribution was deemed a better fit. Neither exponential or Poisson showed a better fit to any of 40 outdegree distributions. For indegree, 2 distributions were unlikely drawn from a power law distribution: one for all age group, and one for heifer age group. Either log normal, exponential nor Poisson distribution showed a better fit to any of 40 indegree distributions. Overall, dairy cattle movement networks in New Zealand between 2000 and 2010 seemed to show a property of a power law distribution.

The majority of farms had an indegree of less than 6. For instance, the proportion of farms that received from less than 6 farms were 98.4%, 98.3%, and 93.1% for calf, heifer, and adult, respectively in 2000. When all age groups were aggregated, this percentage was 90.0%. The corresponding percentages for 2001 and 2002 were as follows: 98.8, 98.7, 94.0, and 91.1 for 2001, and 98.8, 98.4, 95.0, and 92.4 for 2002. On the other hand, a very small proportion of farms had an extremely large outdegree. The percentage of farms that received from greater than 29 farms were 0.09%, 0.07%, and 0.61% for calf, heifer, and adult, respectively, in 2000. When all age groups were aggregated, this percentage was 0.84%. Even smaller proportions were observed for 2001 and 2002; the corresponding percentages were 0.07, 0.02, 0.41, and 0.57 for 2001 and 0.10, 0.14, 0.21, and 0.40 for 2002.

The same statistics were obtained for outdegree. The percentage of farms that sent animals to less than 6 farms were 99.3%, 98.2%, and 95.3%, for calf, heifer, and adult, respectively, in 2000. When all age groups were aggregated, the corresponding percentage was 91.4%. Similar percentages were observed for 2001 and 2002; the corresponding percentages were 99.2, 97.9, 95.9, and 92.3 for calf, heifer, adult, and all age groups together, respectively for 2001, and 99.4, 97.4, 96.1, and 93.3 for 2002. A very small proportion of farms sent animals to a large number of farms. Only 0.03%, 0.07%, 0.31% of farms sent at least one animal to more than 29 farms for calf, heifer, and adult, respectively, in 2000. The percentage aggregated over all age groups was 0.46%. The corresponding percentages were 0.07, 0.11, 0.4, and 0.61 for 2001, and 0, 0.01, 0.3, and 0.45 for 2002.
