# Supplementary material for: Assessing biases in phylodynamic inferences in the presence of super-spreaders
Source: Vet Res. 2019 Sep 27;50:74. doi: 10.1186/s13567-019-0692-5 (PMC6764146; doi:10.1186/s13567-019-0692-5)
Supplement: Supplementary file 6 — Additional file 6. Simulation parameter values. This file provides parameter values used in the disease simulation. [file 13567_2019_692_MOESM6_ESM.docx]

**Additional file 6 Parameter values used in the disease simulation.**

| Parameter | Distribution |
| --- | --- |
| $\boldsymbol{C}_{\boldsymbol{e}}$ (number of effective contacts) | 5 / day (fixed) |
| Latent period (from E to I) | Uniform (0, 100) days |
| Infectious period (from I to R) | Uniform (60, 300) days |
| Substitution rate | 0.012 /site/year (fixed) |
| Baseline probability of detection (P_base_) | 0.01 / day (fixed) |
| Elevated probability of detection (P_inc_) | 0.02 / day (fixed) |
