# Supplementary material for: Assessing biases in phylodynamic inferences in the presence of super-spreaders
Source: Vet Res. 2019 Sep 27;50:74. doi: 10.1186/s13567-019-0692-5 (PMC6764146; doi:10.1186/s13567-019-0692-5)
Supplement: Supplementary file 8 — Additional file 8. The BEAST setting for Birth–death Skyline plot model. This file provides a detailed explanation on how BDSKY model was set. [file 13567_2019_692_MOESM8_ESM.docx]

**Additional file 8** **The BEAST settings for Birth-death Skyline (BDSKY) plot model**

Note that BDSKY can infer not only the tree height but also the origin of the epidemic, which is the start of the outbreak when there was only one infected individual. To compare with the Extended Bayesian Skyline plot model, we used the tree height as a parameter of interest. In our simulation framework, the difference between the origin of the epidemic and the time when the first infection occurred (the tree root) should be negligible because the infection spreads within a herd as soon as the first animal was infected. Nevertheless, we note that the difference between the BEAST estimates on the origin and tree height were sometimes non-trivial. Followings are the prior settings.

- Re (effective reproduction number): dimension set to 3. Prior set log-normal and median 0 (corresponds to 1 in real scale) and variance 1.25 (corresponds to 5%tile being 0.128 and 95% tile being 7.82).
- The becoming noninfectious rate: inverse of period an individual is infectious. For our FMD-like example, this period corresponds to min_S3toS4_FMD (=60) max_S3toS4_FMD (=300), so the average is 180 days, which gives a rate 1/180 = 0.006 (range: 1/300 = 0.003 to 1/60 = 0.017). We therefore used a log-normal distribution with log-scale mean -5.1 (gives median 0.006) and standard deviation 0.7. This translates into infectious period falling into 5%tile 52 and 95%tile 519 days.
- Sampling proportion: dimension to 2. The first dimension was set to be 0 because there was no sampling until around the first sample was collected by design. For the second dimension, the prior was defined as follows. We are sampling one individual per farm. Conservatively assuming that herd size ranges between 20 and 400, the sampling proportion in infected farm ranges from 0.0025 to 0.05. Now we assume that proportion of infected farms sampled is uniform between 0.004 (assuming 1 out of 250 farms sampled, which is very conservative low estimate) and 1. Therefore, the sampling proportion is ranges from 0.004 × 0.0025 = 0.00001 to 1 × 0.05 = 0.05. The initial value for the second dimension was set to be 0.00001.
- Origin of the epidemic: We used uniform prior with limits 0 to 2500, with the initial value being 1500.
- Clock rate: as described in the manuscript. Uniform 0.00002 (2.0E-5) and 0.00005 (5.0E-5) as lower and upper limit, respectively (per site per day). The initial value was set to be 3.0E-5.
- MCMC setting: chain length 50 000 000, store every 1000. When MCMC did not converge till 1×10^8^, we reset Re dimension to 2.
- XML files used can be reproduced by running R code which can be found from [52].
