# Supplementary material for: Assessing biases in phylodynamic inferences in the presence of super-spreaders
Source: Vet Res. 2019 Sep 27;50:74. doi: 10.1186/s13567-019-0692-5 (PMC6764146; doi:10.1186/s13567-019-0692-5)
Supplement: Supplementary file 11 — Additional file 11. Farm characteristics of super-spreaders identified in 100 simulations. This file provides a detailed explanation on super-spreader farms identified in this study. [file 13567_2019_692_MOESM11_ESM.docx]

**Additional file 11** **Characteristics of super-spreaders**

In 100 simulated outbreaks, there were a total of 1953 farms that were infected in at least one outbreak. Among these farms, 18 farms were identified to have infected 20 or more other farms in at least one outbreak. Here, we briefly describe the characteristics of these farms.

- Region: Out of these 18 farms, 14 were from Waikato region. Two farms were from Taranaki region. One farm was each from Manawatu and Canterbury region.
- Degree: See Supplementary Figure 4. There is a large variation in the outdegree (in terms of both animal and farm-level outdegree) among these 18 farms. The median values calculated over 2000 to 2004 for outdegree (animal-level), outdegree (farm-level), indegree (animal-level), and indegree (farm-level) were 19, 2, 19, and 2, respectively. While some of 18 farms (e.g. farm C, D, and P) had consistently high outdegree values (particularly for adult animals), other farms had high outdegree only in one year.
- Herd size and age structure: See Supplementary Figure 5. The boxplots show the distribution of the number of animals present as of 2000 in each super-spreader herd. The red dashed lines indicate the median value for each age category calculated using all farms that had at least one animal in 2000. This shows super-spreader herds are unlikely to have a substantially different herd size nor age structure compared to other herds. Note that the calculated number of animals, which was used in this simulation work, is likely to be an underestimate of the reality because the used database does not capture animals that did not move even once.
