# Supplementary material for: Assessing biases in phylodynamic inferences in the presence of super-spreaders
Source: Vet Res. 2019 Sep 27;50:74. doi: 10.1186/s13567-019-0692-5 (PMC6764146; doi:10.1186/s13567-019-0692-5)
Supplement: Supplementary file 18 — Additional file 18. Univariable associations between each epidemic characteristic and the percent error of the EBSP model. A table describing the result of the univariable linear regression model [file 13567_2019_692_MOESM18_ESM.docx]

**Additional file 18 Univariable associations between each epidemic characteristic and EBSP percent error.** Coefficients are scaled into a proportion rather than percentage.

| **Variable** |  | **Coefficient** | **SE** | **p** |
| --- | --- | --- | --- | --- |
| ***Variables related to super-spreader*** |  |  |  |  |
| **Average number of effective reproduction number (R)** |  | 0.1383 | 0.1425 | 0.3 |
|  |  |  |  |  |
| **Standard deviation of R** |  | 0.01434 | 0.01352 | 0.3 |
|  |  |  |  |  |
| **Max R divided by the total number of infected farms** |  | 0.19071 | 0.12054 | 0.12 |
|  |  |  |  |  |
| **Max R except Index farm divided by the total number of infected farms** |  | 0.03432 | 0.11251 | 0.76 |
|  |  |  |  |  |
| **Presence of a super spreader** | R>40 | 0.032961 | 0.042814 | 0.4 |
|  | R>30 | 0.006264 | 0.023559 | 0.8 |
|  | R>20 | 0.01679 | 0.01691 | 0.3 |
|  | R>15 | 0.0252 | 0.01819 | 0.17 |
|  | R>10 | -0.04106 | 0.042 | 0.34 |
|  |  |  |  |  |
| **Presence of a super spreader except index farm** | R>40 | 0.032961 | 0.042814 | 0.4 |
|  | R>30 | 0.023783 | 0.032894 | 0.47 |
|  | R>20 | 0.0008918 | 0.0235669 | 0.97 |
|  | R>15 | 0.007574 | 0.01732 | 0.66 |
|  | R>10 | -0.01501 | 0.01798 | 0.41 |
| ***Variables related to other epidemic characteristics*** |  |  |  |  |
| **Inclusion of a sample from index farm** | No | Ref |  |  |
|  | Yes | 0.019 | 0.019 | 0.3 |
|  |  |  |  |  |
| **Average path lengths between all infected farms** |  | -0.03425 | 0.0161 | 0.036 |
|  |  |  |  |  |
| **Average path lengths between all sampled farms** |  | -0.03607 | 0.01549 | 0.02 |
|  |  |  |  |  |
| **Average path lengths from the index farm to all infected farms** |  | -0.01786 | 0.0182 | 0.33 |
|  |  |  |  |  |
| **Average path lengths from the index farm to all sampled farms** |  | -0.02126 | 0.01812 | 0.24 |
|  |  |  |  |  |
| **Epidemic duration (day)** |  | -0.0001 | -0.00003 | 0.007 |
|  |  |  |  |  |
| **Number of infected farms** |  | -0.0001069 | 0.0002527 | 0.67 |
|  |  |  |  |  |
| **Proportion of infected farms sampled** |  | -0.17 | 0.14 | 0.23 |
|  |  |  |  |  |
| **Normalised Sackin index** |  | -0.00102 | 0.006105 | 0.86 |
