# Supplementary material for: Assessing biases in phylodynamic inferences in the presence of super-spreaders
Source: Vet Res. 2019 Sep 27;50:74. doi: 10.1186/s13567-019-0692-5 (PMC6764146; doi:10.1186/s13567-019-0692-5)
Supplement: Supplementary file 19 — Additional file 19. Univariable associations between each epidemic characteristic and the percent error of the BDSKY model. A table describing the result of the univariable linear regression model. [file 13567_2019_692_MOESM19_ESM.docx]

**Additional file 19 Univariable associations between each epidemic characteristic and BDSKY percent error.** Coefficients are scaled into a proportion rather than percentage.

| **Variable** |  | **Coefficient** | **SE** | **p** |
| --- | --- | --- | --- | --- |
| ***Variables related to super-spreader*** |  |  |  |  |
| **Average number of effective reproduction number (R)** |  | 0.1885 | 0.19 | 0.33 |
|  |  |  |  |  |
| **Standard deviation of R** |  | 0.05009 | 0.01773 | 0.005 |
|  |  |  |  |  |
| **Max R divided by the total number of infected farms** |  | 0.51874 | 0.15697 | 0.001 |
|  |  |  |  |  |
| **Max R except Index farm divided by the total number of infected farms** |  | -0.08545 | 0.15236 | 0.576 |
|  |  |  |  |  |
| **Presence of a super spreader** | R>40 | -0.02424 | 0.05816 | 0.678 |
|  | R>30 | 0.05264 | 0.03150 | 0.1 |
|  | R>20 | 0.06043 | 0.02222 | 0.008 |
|  | R>15 | 0.04793 | 0.02442 | 0.05 |
|  | R>10 | 0.01734 | 0.05819 | 0.77 |
|  |  |  |  |  |
| **Presence of a super spreader except index farm** | R>40 | -0.02424 | 0.06 | 0.68 |
|  | R>30 | 0.014 | 0.044 | 0.76 |
|  | R>20 | -0.001473 | 0.032 | 0.96 |
|  | R>15 | 0.004956 | 0.023497 | 0.83 |
|  | R>10 | -0.03334 | 0.024 | 0.17 |
| ***Variables related to other epidemic characteristics*** |  |  |  |  |
| **Inclusion of a sample from index farm** | No | Ref |  |  |
|  | Yes | 0.04 | 0.025 | 0.09 |
|  |  |  |  |  |
| **Average path lengths between all infected farms** |  | -0.04075 | 0.0219 | 0.07 |
|  |  |  |  |  |
| **Average path lengths between all sampled farms** |  | -0.03966 | 0.0212 | 0.06 |
|  |  |  |  |  |
| **Average path lengths from the index farm to all infected farms** |  | -0.0478 | 0.024 | 0.05 |
|  |  |  |  |  |
| **Average path lengths from the index farm to all sampled farms** |  | -0.04892 | 0.024 | 0.04 |
|  |  |  |  |  |
| **Epidemic duration (day)** |  | -0.0001007 | 0.0000515 | 0.05 |
|  |  |  |  |  |
| **Number of infected farms** |  | -8.393e-06 | 3.429e-04 | 0.98 |
|  |  |  |  |  |
| **Proportion of infected farms sampled** |  | -0.04064 | 0.20 | 0.84 |
|  |  |  |  |  |
| **Normalised Sackin index** |  | -0.02121 | 0.016 | 0.18 |
