# Supplementary material for: Assessing biases in phylodynamic inferences in the presence of super-spreaders
Source: Vet Res. 2019 Sep 27;50:74. doi: 10.1186/s13567-019-0692-5 (PMC6764146; doi:10.1186/s13567-019-0692-5)
Supplement: Supplementary file 20 — Additional file 20. Univariable associations between each epidemic characteristic and the HPD size of the EBSP model. A table describing the result of the univariable linear regression model. [file 13567_2019_692_MOESM20_ESM.docx]

**Additional file 20 Univariable associations between each epidemic characteristic and EBSP HPD size.** Coefficients are scaled into a proportion rather than percentage.

| **Variable** |  | **Coefficient** | **SE** | **p** |
| --- | --- | --- | --- | --- |
| ***Variables related to super-spreader*** |  |  |  |  |
| **Average number of effective reproduction number (R)** |  | 0.1622 | 0.3 | 0.59 |
|  |  |  |  |  |
| **Standard deviation of R** |  | 0.07645 | 0.027 | 0.006 |
|  |  |  |  |  |
| **Max R divided by the total number of infected farms** |  | 0.82409 | 0.24136 | 0.0009 |
|  |  |  |  |  |
| **Max R except Index farm divided by the total number of infected farms** |  | -0.04361 | 0.23539 | 0.85 |
|  |  |  |  |  |
| **Presence of a super spreader** | R>40 | 0.31879 | 0.08 | 0.0002 |
|  | R>30 | 0.08474 | 0.04854 | 0.08 |
|  | R>20 | 0.08657 | 0.03446 | 0.01 |
|  | R>15 | 0.045 | 0.03813 | 0.24 |
|  | R>10 | 0.07303 | 0.09 | 0.42 |
|  |  |  |  |  |
| **Presence of a super spreader except index farm** | R>40 | 0.31879 | 0.08 | 0.0002 |
|  | R>30 | 0.187 | 0.06 | 0.006 |
|  | R>20 | 0.02539 | 0.05 | 0.61 |
|  | R>15 | -0.01802 | 0.04 | 0.62 |
|  | R>10 | -0.06806 | 0.037 | 0.07 |
| ***Variables related to other epidemic characteristics*** |  |  |  |  |
| **Inclusion of a sample from index farm** | No | Ref |  |  |
|  | Yes | 0.003599 | 0.039642 | 0.93 |
|  |  |  |  |  |
| **Average path lengths between all infected farms** |  | -0.1085 | 0.03264 | 0.001 |
|  |  |  |  |  |
| **Average path lengths between all sampled farms** |  | -0.11602 | 0.03 | 0.0003 |
|  |  |  |  |  |
| **Average path lengths from the index farm to all infected farms** |  | -0.06914 | 0.04 | 0.07 |
|  |  |  |  |  |
| **Average path lengths from the index farm to all sampled farms** |  | -0.08076 | 0.04 | 0.03 |
|  |  |  |  |  |
| **Epidemic duration (day)** |  | -0.0006 | 0.00005 | <0.00001 |
|  |  |  |  |  |
| **Number of infected farms** |  | 0.0006551 | 0.0005249 | 0.22 |
|  |  |  |  |  |
| **Proportion of infected farms sampled** |  | -1.0685 | 0.28 | 0.000252 |
|  |  |  |  |  |
| **Normalised Sackin index** |  | -0.04724 | 0.02 | 0.05 |
