# Supplementary material for: Assessing biases in phylodynamic inferences in the presence of super-spreaders
Source: Vet Res. 2019 Sep 27;50:74. doi: 10.1186/s13567-019-0692-5 (PMC6764146; doi:10.1186/s13567-019-0692-5)
Supplement: Supplementary file 21 — Additional file 21. Univariable associations between each epidemic characteristic and the HPD size of the BDSKY model. A table describing the result of the univariable linear regression model. [file 13567_2019_692_MOESM21_ESM.docx]

**Additional file 21 Univariable associations between each epidemic characteristic and EBSP HPD size.** Coefficients are scaled into a proportion rather than percentage.

| **Variable** |  | **Coefficient** | **SE** | **p** |
| --- | --- | --- | --- | --- |
| ***Variables related to super-spreader*** |  |  |  |  |
| **Average number of effective reproduction number (R)** |  | -0.1226 | 0.23 | 0.59 |
|  |  |  |  |  |
| **Standard deviation of R** |  | 0.05300 | 0.02 | 0.015 |
|  |  |  |  |  |
| **Max R divided by the total number of infected farms** |  | 0.89914 | 0.17572 | <0.0001 |
|  |  |  |  |  |
| **Max R except Index farm divided by the total number of infected farms** |  | 0.3123 | 0.1797 | 0.08 |
|  |  |  |  |  |
| **Presence of a super spreader** | R>40 | 0.16617 | 0.07 | 0.016 |
|  | R>30 | 0.02164 | 0.03812 | 0.57 |
|  | R>20 | 0.03027 | 0.027 | 0.27 |
|  | R>15 | 0.03948 | 0.029 | 0.18 |
|  | R>10 | 0.05436 | 0.069 | 0.44 |
|  |  |  |  |  |
| **Presence of a super spreader except index farm** | R>40 | 0.16617 | 0.07 | 0.016 |
|  | R>30 | 0.082 | 0.05 | 0.124 |
|  | R>20 | 0.03586 | 0.04 | 0.35 |
|  | R>15 | 0.01873 | 0.028 | 0.51 |
|  | R>10 | -0.04578 | 0.029 | 0.12 |
|  |  |  |  |  |
| ***Variables related to other epidemic characteristics*** |  |  |  |  |
| **Inclusion of a sample from index farm** | No | Ref |  |  |
|  | Yes | 0.033 | 0.03 | 0.27 |
|  |  |  |  |  |
| **Average path lengths between all infected farms** |  | -0.10773 | 0.024 | 0.00002 |
|  |  |  |  |  |
| **Average path lengths between all sampled farms** |  | -0.11311 | 0.023 | <0.00001 |
|  |  |  |  |  |
| **Average path lengths from the index farm to all infected farms** |  | -0.06746 | 0.028 | 0.02 |
|  |  |  |  |  |
| **Average path lengths from the index farm to all sampled farms** |  | -0.07666 | 0.029 | 0.008 |
|  |  |  |  |  |
| **Epidemic duration (day)** |  | -0.0004 | 0.00004 | <0.00001 |
|  |  |  |  |  |
| **Number of infected farms** |  | -0.0002252 | 0.0004093 | 0.58 |
|  |  |  |  |  |
| **Proportion of infected farms sampled** |  | -0.6437 | 0.22 | 0.005 |
|  |  |  |  |  |
| **Normalised Sackin index** |  | -0.009738 | 0.02 | 0.6 |
