# Supplementary material for: Assessing biases in phylodynamic inferences in the presence of super-spreaders
Source: Vet Res. 2019 Sep 27;50:74. doi: 10.1186/s13567-019-0692-5 (PMC6764146; doi:10.1186/s13567-019-0692-5)
Supplement: Supplementary file 22 — Additional file 22. Results of the multivariable models. A table describing the results of the final multivariable linear regression model for each statistic. [file 13567_2019_692_MOESM22_ESM.docx]

**Additional file 22 Final multivariable models showing the coefficients of each super-spreader and epidemic characteristic.** Coefficients are scaled into a proportion rather than percentage.

| **Variable** |  | **Coefficient** | **SE** | **p** |
| --- | --- | --- | --- | --- |
| ***Model: EBSP percent error*** |  |  |  |  |
| **Epidemic duration (day)** |  | -0.0001 | -0.00003 | 0.007 |
|  |  |  |  |  |
|  |  |  |  |  |
| ***Model: BDSKY percent error*** |  |  |  |  |
| **Max R divided by the total number of infected farms** |  | 0.51874 | 0.15697 | 0.001 |
|  |  |  |  |  |
| ***Model: EBSP HPD size*** |  |  |  |  |
| **Epidemic duration (day)** |  | -0.0005 | 0.00006 | <0.00001 |
| **Proportion of infected farms sampled** |  | -0.38 | 0.22 | 0.09 |
|  |  |  |  |  |
| ***Model: BDSKY HPD size*** |  |  |  |  |
| **Max R divided by the total number of infected farms** |  | 0.43 | 0.144 | 0.003 |
| **Epidemic duration (day)** |  | -0.0004 | 0.00005 | <0.00001 |
